# Modeling Versus Balancing Approaches to Addressing Instrumental Variables in Weighting: A Comparison of the Outcome‐Adaptive Lasso, Stable Balancing Weighting, and Stable Confounder Selection

**DOI:** 10.1002/pds.70173

**Published:** 2025-06-27

**Authors:** Byeong Yeob Choi, M. Alan Brookhart

**Affiliations:** ^1^ Department of Population Health Sciences UT Health San Antonio San Antonio Texas USA; ^2^ Department of Population Health Sciences Duke University Durham North Carolina USA

**Keywords:** instrumental variable, outcome‐adaptive lasso, positivity, propensity score, stable balancing weighting, stable confounder selection

## Abstract

**Background:**

Variable selection is essential for propensity score (PS)‐weighted estimators. Recent work shows that including instrumental variables (IVs), associated with only treatment but not with the outcome, can impact both the bias and precision of the PS‐weighted estimators.

**Methods:**

The outcome‐adaptive lasso (OAL) is an innovative model‐based method adapting the popular adaptive lasso variable selection to causal inference. It attempts to identify IVs, so one can exclude them from the PS model. Unlike the model‐based approach, stable balancing weighting (SBW) estimates inverse probability weights directly while minimizing the variance of the weights and covariate imbalance simultaneously. Based on its variance optimization algorithm, SBW may provide some protection against the impact of IVs. Lastly, we considered stable confounder selection (SCS), which assesses the stability of model‐based effect estimates.

**Results:**

The authors present the results of simulation studies to investigate which method performs the best when moderate or strong IVs are used. The simulation studies consider IVs and spurious variables to generate extreme PSs. In simulations, SBW generally outperformed OAL and SCS in terms of reducing mean squared error, notably when the IVs were strong, and many covariates were highly correlated. Our empirical application to the effect of abciximab treatment demonstrates that SBW is a robust method to effectively handle limited overlap.

**Conclusions:**

Our numerical results support the use of SBW in situations where IVs or near‐IVs may lead to practical violations of positivity assumptions.

**Summary**

- The use of instrumental variables (IVs) reduces the accuracy of augmented inverse‐probability‐of‐treatment weighted (AIPTW) estimators.
- Outcome‐adaptive lasso (OAL) and stable balancing weighting (SBW) are model‐ and balancing‐based approaches that help mitigate the impact of IVs.
- The stable confounder selection (SCS) method is a model‐based approach designed to assess the stability of AIPTW estimators.
- Simulation results demonstrate that SBW generally outperforms OAL and SCS notably when IVs are strong and many covariates are highly correlated.
- SBW effectively controls the influence of IVs on AIPTW estimators.

## 1 Introduction

### 1.1 Mitigating the Impact of Extreme Propensity Scores

Propensity score (PS) methods are an approach for estimating treatment effects in the presence of confounding [1]. A PS is defined as the conditional probability of treatment given all measured confounders. Under the assumptions of no unmeasured confounding (conditional exchangeability), consistency, positivity, and correct PS model specification, various statistical techniques, such as weighting, matching, subclassification, and regression, can be used to estimate the treatment effects [1]. Among those, PS weighting has good theoretical properties in that the resulting effect estimators are consistent for target causal estimands that generalize to well‐defined populations [2]. However, the PS‐weighted estimators are sensitive to extreme PSs and practical or near violations of positivity. Under lack of overlap, PS‐weighted estimators and even regression estimators could be more sensitive to model misspecification [3, 4, 5].

Mitigating extreme PSs or positivity violations is necessary to obtain reliable PS‐weighted estimators. Particularly, extreme PSs inflate the variances of the inverse‐probability‐of‐treatment weighted (IPTW) and augmented IPTW (AIPTW) estimators, which are the standard estimators for the average treatment effect (ATE) [2, 6]. Many studies have demonstrated that excluding instrumental variables (IVs) or IV‐like variables from the adjustment set can help prevent extreme PSs or avoid bias amplification [7, 8, 9, 10, 11, 12]. However, in practice, it is hard to identify such variables a priori, and therefore, data‐adaptive variable selection might be useful. Based on the principle that the impact of IVs must be minimized but outcome predictors should be included in the adjustment set for efficiency gains [7], Shortreed and Ertefaie [13] proposed the outcome‐adaptive lasso (OAL), where the outcome‐covariate associations are used to tune the lasso PS model. Their theoretical and numerical investigations demonstrated that OAL effectively chose true confounders and outcome predictors and performed similarly or better than existing variable selection methods [14, 15].

Another PS approach for ATE that can handle extreme PSs is stable balancing weighting (SBW) proposed by Zubizarreta [16]. Unlike standard PS methods that estimate the PSs based on maximum likelihood (ML) and invert them to generate inverse probability weights, SBW directly calculates the weights as minimizing the variance of the weights while approximately balancing covariates. Therefore, SBW does not require a specific PS model but the functions of the covariates to be approximately balanced. Wang and Zubizarreta [17] demonstrated that under some regularity conditions, the PS weights estimated by SBW converge in probability to the true inverse probability weights. Even though SBW was not designed to control the effects of IVs on the PSs, it is expected to reduce the impacts of IVs because the solution to SBW aims to minimize the variance of the weights.

There are some differences between OAL and SBW. OAL specifies a functional form of the PS model. Then, it identifies specific variables that need to be included (true confounders and outcome predictors) in the model using the adaptive lasso [18]. In contrast, SBW directly estimates PS weights, without specifying the PS model, as minimizing their variance while approximately balancing the functions of covariates. Despite these differences, both OAL and SBW commonly use a single set of covariates. A different approach is to explicitly assess the stability of the treatment effect estimator across different covariate subsets as a criterion for choosing control variables. Loh and Vansteelandt [19] proposed such a method, which employs the outcome and PS models to order the covariates based on their strength of associations and evaluates the stability of the effect estimates based on the series of nested covariate sets. The final estimate is chosen as the most stable one. Hereafter, we will call this method stable confounder selection (SCS).

### 1.2 The Objective of This Study

In this study, we compared the OAL, SCS, and SBW, three promising statistical methods for estimating the ATE in the presence of extreme PSs. To compare these three methods, we adapted the simulation experiments of Shortreed and Ertefaie [13], where a large number of covariates are examined, including IVs and spurious covariates. We modified their simulations to create extreme PSs and heterogeneous treatment effects. Performance was measured based on bias, mean squared errors (MSEs), Monte Carlo standard errors, and confidence interval coverage rates.

### 1.3 Assumptions on Covariates

The methods discussed assume that the covariate set under consideration includes all necessary confounding variables to control for confounding bias while excluding “bad controls”—variables affected by treatment [20]. “Bad controls” include intermediate variables on the causal path from treatment to outcome and colliders, which are common effects of both treatment and outcome. Adjusting for these variables can introduce bias in treatment effect estimation. Other examples of “bad controls” include IVs and spurious variables outside the causal system of interest [20, 21]. While these variables do not contribute to bias reduction, they can impact estimation precision. The optimal approach is to use causal knowledge to exclude unnecessary variables from the analysis [22]. Throughout this paper, we assume that “bad controls”, such as intermediate variables or colliders, can be identified and excluded using causal knowledge. The data‐adaptive methods compared in this study can aid in selecting necessary covariates, such as outcome‐predictors, and managing unnecessary variables like IVs and spurious variables.

We applied these methods to data on the effect of abciximab on 6‐month mortality among patients who underwent percutaneous coronary intervention (PCI). The dataset included seven patient and procedural characteristics (three numeric and four binary variables). The numeric variables included height (108 cm to 196 cm), left ventricular ejection fraction (LVEF) (0% to 90%), and number of vessels involved in the patient's initial PCI procedure (NVIP) (0 to 5). The binary covariates included coronary stent deployment, female gender, diabetes mellitus diagnosis (DMD), and acute myocardial infarction (AMI) within the previous 7 days.

All covariates were measured before abciximab administration, ensuring they were not intermediate variables. However, even pre‐treatment variables can serve as colliders if influenced by unmeasured common causes of those variables and treatment or outcome, leading to collider bias [20, 23, 24, 25]. In our case, the unmeasured common causes may include socio‐economic status (SES) and frailty. Notably, female gender is unlikely to be an effect of these unmeasured factors and therefore not a collider. Additionally, LVEF, NVIP, stent placement, DMD, and AMI are strong confounders for the effect of abciximab on 6‐month mortality, as they influence both treatment assignment and mortality risk. Thus, adjusting for them should reduce bias rather than introduce it. Height, while not a direct predictor of abciximab administration, may influence mortality due to baseline differences in health risks. It is reasonable to assume that height is an effect of SES but not frailty, implying that adjusting for it does not induce collider bias.

Expanding the covariate set with interaction terms and squared terms for continuous variables can help mitigate residual confounding. However, our analysis demonstrated that this exacerbated the issue of limited overlap. We evaluated how effectively OAL, SBW, and SCS handle these extended variables, where some of them could be IVs or spurious variables, in this real‐world application.

## 2 Methods

### 2.1 Notation, Assumptions, and AIPTW Estimator

Suppose we are interested in estimating the ATE from a sample of n subjects. For unit i, let $Z_{i}$ be the treatment indicator labeled one if receiving the active treatment and 0 if receiving the control. Let $Y_{i}\left(z\right)$ be the potential outcome that would have been observed if the treatment level of unit i had been set to $z\in \left\{0,1\right\}$. Under the Stable Unit Treatment Value Assumption (SUTVA), the observed outcome can be expressed as $Y_{i}=Z_{i}Y_{i}\left(1\right)+\left(1-Z_{i}\right)Y_{i}\left(0\right)$. Let $X_{i}=\left(X_{i1},\ldots ,X_{\mathrm{ij}},\ldots ,X_{\mathrm{iJ}}\right)$ denote a vector of J covariates. Then, the PS is defined as the probability of getting treatment conditional on all observed confounders $X_{i}$: $e_{i}=e\left(X_{i}\right)=\mathrm{Pr}\left(Z_{i}=1|X_{i}\right)$. We denote by $E\left(Y_{i}|Z_{i}=z,X_{i}\right)$ the expected value of the outcome for the group $Z_{i}=z$ conditional on $X_{i}$.

We make two additional assumptions for PS‐weighted analysis. The first assumption is that the potential outcomes are independent of treatment assignment conditional on the observed confounders. In other words, $\left\{Y_{i}\left(1\right),Y_{i}\left(0\right)\right\}⊥Z_{i}|X_{i}$, where ⊥ indicates statistical independence. The second assumption is that all PSs are strictly between 0 and 1: $0<e_{i}<1$ for all i=1,2,…,n. This is called positivity.

Our target parameter ATE is expressed as $\tau =E\left\{Y_{i}\left(1\right)-Y_{i}\left(0\right)\right\}$. Let ${\hat{e}}_{i}$ and $\hat{E}\left(Y_{i}|Z_{i}=z,X_{i}\right)$ denote the estimated PS and expected value of the outcome for subject i, respectively. Define the inverse probability of treatment weights as

$${\hat{W}}_{i}=\frac{1}{Z_{i}{\hat{e}}_{i}+\left(1-Z_{i}\right)\left(1-{\hat{e}}_{i}\right)}.$$

Then, the AIPTW estimator for the ATE can be written as

(1) $$\begin{array}{cc} \hat{\tau }= & n^{-1}\sum_{i=1}^{n}\left(2Z_{i}-1\right){\hat{W}}_{i}\left\{Y_{i}-\hat{E}\left(Y_{i}|Z_{i},X_{i}\right)\right\}\\ & +\hat{E}\left(Y_{i}|Z_{i}=1,X_{i}\right)-\hat{E}\left(Y_{i}|Z_{i}=0,X_{i}\right). \end{array}$$

The AIPTW estimator in Equation (1) is consistent for the ATE if either the PS model or the outcome model is correctly specified. When the PS and outcome models are correctly specified, then the influence function for subject i is defined as

$$\begin{array}{cc} {\hat{\psi }}_{i}= & \left(2Z_{i}-1\right){\hat{W}}_{i}\left\{Y_{i}-\hat{E}\left(Y_{i}|Z_{i},X_{i}\right)\right\}\\ & +\hat{E}\left(Y_{i}|Z_{i}=1,X_{i}\right)-\hat{E}\left(Y_{i}|Z_{i}=0,X_{i}\right)-\hat{\tau }. \end{array}$$

Then, the asymptotic variance of $\hat{\tau }$ is estimated by the sample variance of ${\hat{\psi }}_{i}$. Use of AIPTW is essential for our study, because data‐adaptive approaches such as lasso give valid inference results when in conjunction with doubly robust estimators including AIPTW and targeted ML estimation estimators [26, 27, 28].

### 2.2 Outcome‐Adaptive Lasso (OAL)

Shortreed and Ertefaie [13] extended the adaptive lasso [18] to variable selection for causal inference. Traditional lasso focuses on selecting variables to optimize the prediction of treatment assignment, which may overlook important outcome‐related covariates that are not helpful for prediction. OAL addresses this limitation by incorporating information from both the outcome model and the PS model. The process begins by fitting an outcome model using all covariates. Then, OAL assigns adaptive weights to each covariate, giving greater priority to those that have large absolute outcome model coefficients. These weighted covariates are included in the PS model through a lasso framework, where the lasso penalty shrinks coefficients for IVs and spurious variables toward zero.

To provide a concrete understanding of OAL, we present its mathematical details. Let C denote a set of covariates associated with both treatment and outcome, P a set of predictors of the outcome but not of treatment, I a set of IVs that are predictive of only treatment, and S a set of covariates not predictive of both treatment and outcome. The ideal PS model includes all true confounders (C) to remove confounding bias and all outcome predictors (P) to increase precision while excluding the IVs and spurious variables.

OAL aims to select the covariates in C and P and estimate the corresponding coefficients by incorporating the outcome–covariate relationship. Specifically, assume a logit model for the PS,

$$e\left(X_{i};\beta_{0},\beta \right)=\frac{e^{\beta_{o}+\beta^{T}X_{i}}}{1+e^{\beta_{o}+\beta^{T}X_{i}}},$$

where $\beta_{0}$ is an intercept and β is a vector of the regression parameters for $X_{i}$. Then, OAL estimates the regression coefficients by maximizing the following penalized log‐likelihood

(2) $$\begin{array}{c} \hat{\beta }={\text{argmax}}_{\beta }\left[\sum_{i=1}^{n}\left\{Z_{i}\left(\beta_{o}+\beta^{T}X_{i}\right)-\log\left(1+e^{\beta_{o}+\beta^{T}X_{i}}\right)\right\}-\lambda_{n}\sum_{j=1}^{J}{\hat{\omega }}_{j}\left|\beta_{j}\right|\right], \end{array}$$

where ${\hat{\omega }}_{j}={\left|{\tilde{\alpha }}_{j}\right|}^{-\gamma }$, γ>1, and ${\tilde{\alpha }}_{j}$ refers to the jth element of $\tilde{\alpha }$, which is the vector for the coefficient estimates for $X_{i}$ in the outcome model. If $|{\tilde{\alpha }}_{j}|$ is small, then a large penalty is given for that covariate. We typically standardize the covariates for the penalty to be meaningful. Theorem 1 of Shortreed and Ertefaie [13] implies that OAL selects those in the covariate sets C and P with probability tending to 1 and that the resulting estimates of the confounders and outcome predictors behave like the ML estimates when the target PS model is known in advance. This theorem holds if $\lambda_{n}/\sqrt{n}\to 0$ and $\lambda_{n}n^{\gamma /2-1}\to \infty$.

OAL has two tuning parameters, $\lambda_{n}$ and γ. In their simulations, Shortreed and Ertefaie considered several values for $\lambda_{n}::\Lambda =\left\{n^{-10},n^{-5},n^{-2},n^{-1},n^{-0.75},n^{-0.5},n^{-0.25},n^{0.25},\text{and}\;n^{0.49}\right\}$. These values satisfy the condition that $\lambda_{n}/\sqrt{n}\to 0$ for Theorem 1 of Shortreed and Ertefaie. Another regularity condition is that $\lambda_{n}n^{\gamma /2-1}\to \infty$. To find such values of γ, they set the equation $\lambda_{n}n^{\gamma /2-1}=n^{2}$ and found γ to satisfy the equation for a given $\lambda_{n}$. Therefore, in this framework, we need to choose only the optimal $\lambda_{n}$ from Λ and γ is followed according to $\lambda_{n}n^{\gamma /2-1}=n^{2}$. Nothing that the inverse probability weights balance covariate means between two treatment groups, Shortreed and Ertefaie proposed to use the following weighted absolute mean difference (wAMD) to find the optimal $\lambda_{n}$

(3) $$\begin{array}{c} \begin{array}{c} \text{wAMD}\left(\lambda_{n}\right)=\sum_{j=1}^{J}\left|{\tilde{\alpha }}_{j}\right|\left|\frac{\sum_{i=1}^{n}\left(Z_{i}X_{\mathrm{ij}}\right)/{\hat{e}}_{i}^{\lambda_{n}}}{\sum_{i=1}^{n}Z_{i}/{\hat{e}}_{i}^{\lambda_{n}}}-\frac{\sum_{i=1}^{n}\left(\left(1-Z_{i}\right)X_{\mathrm{ij}}\right)/\left(1-{\hat{e}}_{i}^{\lambda_{n}}\right)}{\sum_{i=1}^{n}\left(1-Z_{i}\right)/\left(1-{\hat{e}}_{i}^{\lambda_{n}}\right)}\right|, \end{array} \end{array}$$

where ${\hat{e}}_{i}^{\lambda_{n}}$ is the estimated PS for subject i using the regression coefficient estimates obtained by solving Equation (2) with a given $\lambda_{n}$. Noting that ${\tilde{\alpha }}_{j}$ in Equation (3) represents the association between the outcome and covariate $X_{\mathrm{ij}}$, the contribution of $X_{\mathrm{ij}}$ will be minimal to wAMD if the covariate has a negligible association with the outcome.

### 2.3 Stable Balancing Weighting (SBW)

SBW takes a different approach from model‐based methods by directly estimating the inverse probability weights that satisfy specific balancing constraints. It begins by identifying the covariates that need to be balanced. For each covariate, SBW imposes a balance condition, ensuring that the weighted mean in each group aligns with the corresponding mean in the target population. The method then solves an optimization problem to find the set of weights that minimize their variability while approximately balancing the covariates in the control and treatment groups toward the overall means [29]. Specifically, SBW determines the weights for the control group, ensuring that the weighted covariate means are approximately balanced toward the overall means. It then calculates the weights for the treatment group, applying the same balancing principle. Under standard smoothness conditions on the PS function, SBW weights have been shown to provide consistent estimates of the true inverse probability weights [17].

Suppose that we try to find the weights for the control individuals. These weights balance the covariate distribution of the control group toward that of the combined group. Let $B\left(X\right)={\left\{B_{1}\left(X\right),B_{2}\left(X\right),\ldots ,B_{K}\left(X\right)\right\}}^{T}$ denote a vector of K real‐valued functions or transformations of X. In general, K is equal to or larger than J. The SBW weights for the control individuals are obtained by minimizing the following objective function with respect to ω [16, 29]

(4) $$\begin{array}{c} {\mathrm{minimize}}_{\omega }\sum_{i:Z_{i}=0}{\left(\omega_{i}-{\bar{\omega }}_{c}\right)}^{2}, \end{array}$$

subject to

$$\left|\sum_{i:Z_{i}=0}\omega_{i}B_{k}\left(X_{i}\right)-\frac{1}{n}\sum_{i}B_{k}\left(X_{i}\right)\right|\leq \delta_{k},k=1,2,\ldots ,K$$

$$\sum_{i:Z_{i}=0}\omega_{i}=1$$

$$\omega_{i}\geq 0\;\text{for}\;i:Z_{i}=0.$$

Here, ${\bar{\omega }}_{c}$ is the average of the control weights. Based on Equation (4), SBW attempts to minimize the variance of the control weights subject to the given balancing and normalization constraints and positivity. The balancing constraints ensure that the absolute difference between the weighted mean of each $B_{k}\left(X_{i}\right)$ in the control group and the overall sample mean is at most $\delta_{k}$. The estimated weights are used as ${\hat{W}}_{i}$ of the AIPTW estimator for the control group. We can find the weights for the treated individuals in the same way, and those weights are used as ${\hat{W}}_{i}$ of the AIPTW estimator for the treatment group.

Choosing appropriate $\delta_{k}$ is critical for SBW because it determines the degree to which covariates are balanced toward the overall means. Let $S_{k}\left(z\right)$ denote the standard deviation of $B_{k}\left(X\right)$ in group $z\in \left\{0,1\right\}$. We considered $\delta \cdot S_{k}\left(z\right)$ as candidates of $\delta_{k}$, where δ∈ {0.0001, 0.001, 0.002, 0.005, 0.01, 0.02, 0.05, 0.1}. To select the optimal δ, we employed the bootstrap method proposed by Wang and Zubizarreta [17] and Chattopadhyay et al. [29]. For a given δ, SBW weights are first computed for the treated and control groups using the original sample. A bootstrap sample is then drawn, and the standardized absolute distances (ASDs) between the weighted group means and the overall means of each $B_{k}\left(X\right)$ for k=1,…,K are calculated and summed across all covariates. This process is repeated multiple times (e.g., 200 bootstrap samples), and the resulting ASDs are averaged. The optimal δ is selected as the value that minimizes this bootstrap‐averaged ASD.

### 2.4 Stable Confounder Selection (SCS)

Loh and Vansteelandt [19] proposed a confounder selection approach targeting stable treatment effect estimators. The key idea is that once the PS model includes covariates sufficient to control for confounding, adding more covariates not associated with both treatment and outcome should not significantly change the effect estimator. Accordingly, this approach orders the covariates based on the strength of their associations with treatment and with outcome in decreasing priority for confounding adjustment. Then, the AIPTW estimates are calculated using the series of nested covariate sets, and the smallest set that yields the most stable AIPTW estimate is chosen. The stability is examined based on the inverse variance weighted moving average with a fixed window width (see equation 5 of Loh and Vansteelandt). We used a window width of five as in Loh and Vansteelandt. The main features of the outcome‐adaptive lasso, stable balancing weighting, and stable confounder selection are outlined in Table 1.

**TABLE 1:** Features of the outcome‐adaptive lasso, stable balancing weighting, and stable confounder selection.

	Outcome‐adaptive lasso	Stable balancing weighting	Stable confounder selection
Features	Identifies an explicit set of instrumental and spurious variables. It estimates the lasso coefficients of the propensity score model using the outcome–covariates associations as coefficient‐specific weights.	Calculates balancing weights with minimal dispersion while approximately balancing covariates.	Uses covariate–treatment and covariate–outcome associations to prioritize covariates. Assesses the stability of treatment effect estimates based on the inverse variance weighted moving average.
Requirements	Propensity score and outcome models need to be specified. Tuning parameters for lasso penalties need to be chosen.	Balancing criteria need to be specified. Degree of approximate balancing needs to be chosen.	Propensity score and outcome models need to be specified. A window width needs to be chosen for the moving average.
Pros	Selects covariates for accurate causal inference and estimates the propensity scores.	Propensity score models are not needed. Estimated weights have the minimum variance for given balancing criteria.	Prioritizes covariates based on their associations with treatment and outcome. Evaluates the effect estimates across different subsets of covariates.
Cons	Variable selection could be inaccurate if either the propensity score or outcome model is misspecified. It requires parameterizing the conditional associations between each covariate and either treatment or outcome as regression coefficients.	Propensity scores cannot be estimated.	Variable selection could be inaccurate if either the propensity score or outcome model is misspecified. It requires parameterizing the conditional associations between each covariate and either treatment or outcome as regression coefficients.

## 3 Simulation Study

### 3.1 Simulation Design

We conducted simulations to compare OAL, SCS, and SBW in terms of bias, MSE, empirical standard error (EmpSE), average of the estimated standard errors (AvgSE), and 95% confidence interval coverage rate (CovRate) when IVs and spurious variables exist. We adopted the simulation design of Shortreed and Ertefaie with slight modifications. The first step was to simulate J (20 or 40) covariates from a multivariate standard normal distribution with a common correlation coefficient ρ. We considered two correlation structures: no correlation $\left(\rho =0\right)$ and a strong correlation $\left(\rho =0.5\right)$.

For all scenarios, J−6 coefficients were set to zeros in both treatment and outcome models. In other words, J−6 covariates were spurious. However, the first six coefficients were not zeros, with varying magnitudes of the associations with treatment and outcome. The first two covariates, $X_{1}$ and $X_{2}$, were true confounders; the next two covariates, $X_{3}$ and $X_{4}$, were predictors of only the outcome. Finally, the fifth and sixth covariates, $X_{5}$ and $X_{6}$, were predictive of only the treatment, that is, IVs.

We generated a binary treatment, Z, from a Bernoulli distribution with $\text{logit}\left\{\mathrm{Pr}\left(Z=1|X\right)\right\}=\beta_{0}+\beta^{T}X$, where $\beta_{o}=0$ and $\beta ={\left(0.5,0.5,0,0,\theta ,\theta ,0,0,\ldots ,0\right)}^{T}$. The parameter θ determined how strongly the two IVs ($X_{5}$ and $X_{6}$) were associated with treatment and was set to 0.5 or 1 to generate moderate or strong IVs. A continuous outcome Y was generated from the following linear model, $Y=\mathrm{\tau Z}+\alpha_{o}+\alpha^{T}X+\varepsilon$, where $\alpha_{o}=0$, $\alpha =\left(1,1,1,1,0,0,0,0,\ldots ,0\right)$, and ε was a normal random variate with mean 0 and standard deviation 2. Because the mean outcome model implied constant treatment effects, the ATE was equal to τ=1. In addition, we considered heterogeneous treatment effects by replacing τZ with $\left(1+X_{1}+X_{2}\right)Z$, where the treatment effects varied based on $\left(1+X_{1}+X_{2}\right)$. However, the ATE was still equal to 1 because $E\left(1+X_{1}+X_{2}\right)=1$.

In summary, we considered 32 simulation scenarios: two numbers of covariates $\left(J=20\;\text{or}\;40\right)$, two correlation structures (ρ = 0 or 0.5), two degrees of IV strength (θ = 0.5 or 1), constant or heterogeneous treatment effects, and two sample sizes (n = 500 or 2000). Each scenario was simulated 500 times. We mainly presented the simulation results with n=500 in the following subsection. Those with n=2000 are presented in Tables S1–S4.

We considered six types of AIPTW estimators, for which the outcome models were fitted using ordinary least squares and shared the covariates with the PS models. The first type used a logistic PS based on the true confounders and outcome predictors and was referred to as “Target”. This estimator was expected to be optimal in terms of MSE. The second used a logistic PS based on a set of potential confounders ($X_{1},X_{2},\ldots ,X_{6}$), excluding spurious variables but including IVs, and was referred to as “Confounders”. The third used a logistic PS based on all available covariates, including spurious variables, and was referred to as “All covariates”. The fourth was OAL based on a logistic PS, where the coefficients were estimated by maximizing the penalized likelihood in Equation (2). The fifth was SCS with a logistic PS. The last method was SBW. All methods relied on PS models except SBW. Noting that “Target” is a benchmark method, we calculated the relative MSE (R‐MSE) of each method as the MSE divided by that of “Target”.

We used the R package **sbw** [30] to implement SBW and chose its tuning parameter $\left(\delta \right)$ using the bootstrap method (iteration = 200) from a default set. We used the R package **glmnet** [31] to implement OAL with the tuning parameters selected based on $\text{wAMD}\left(\lambda_{n}\right)$. We implemented SCS using the R code available at the GitHub link (https://github.com/wwloh/stability‐confounder‐select).

### 3.2 Simulation Results

**FIGURE 1:**
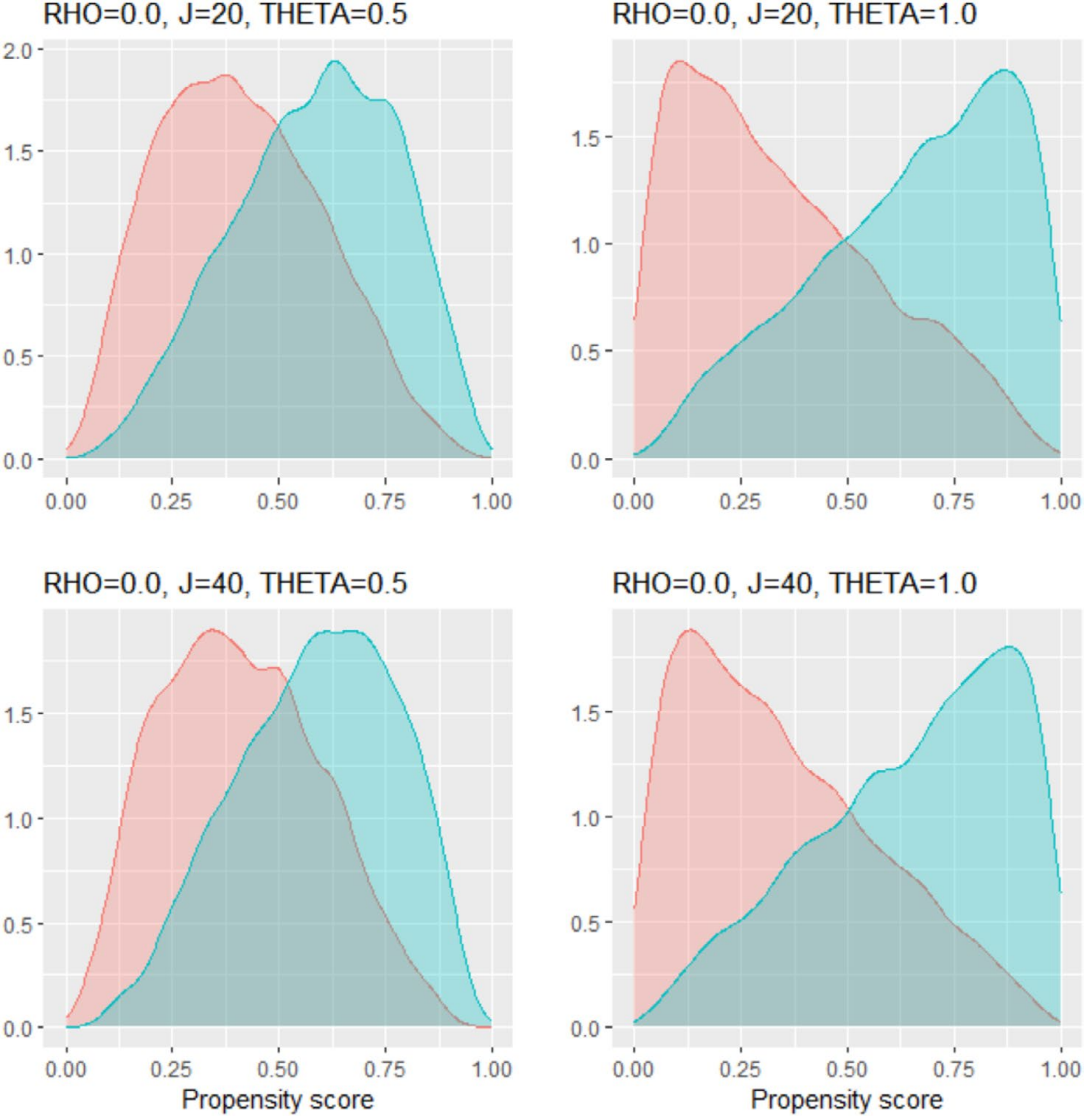
Distributions of the propensity scores across simulation scenarios that differ by the number of covariates (J) and instrument strength (*θ*). The correlation coefficient among the covariates (*ρ*) is fixed at 0. The red and blue density curves represent the propensity scores for the control and treatment groups, respectively.

**FIGURE 2:**
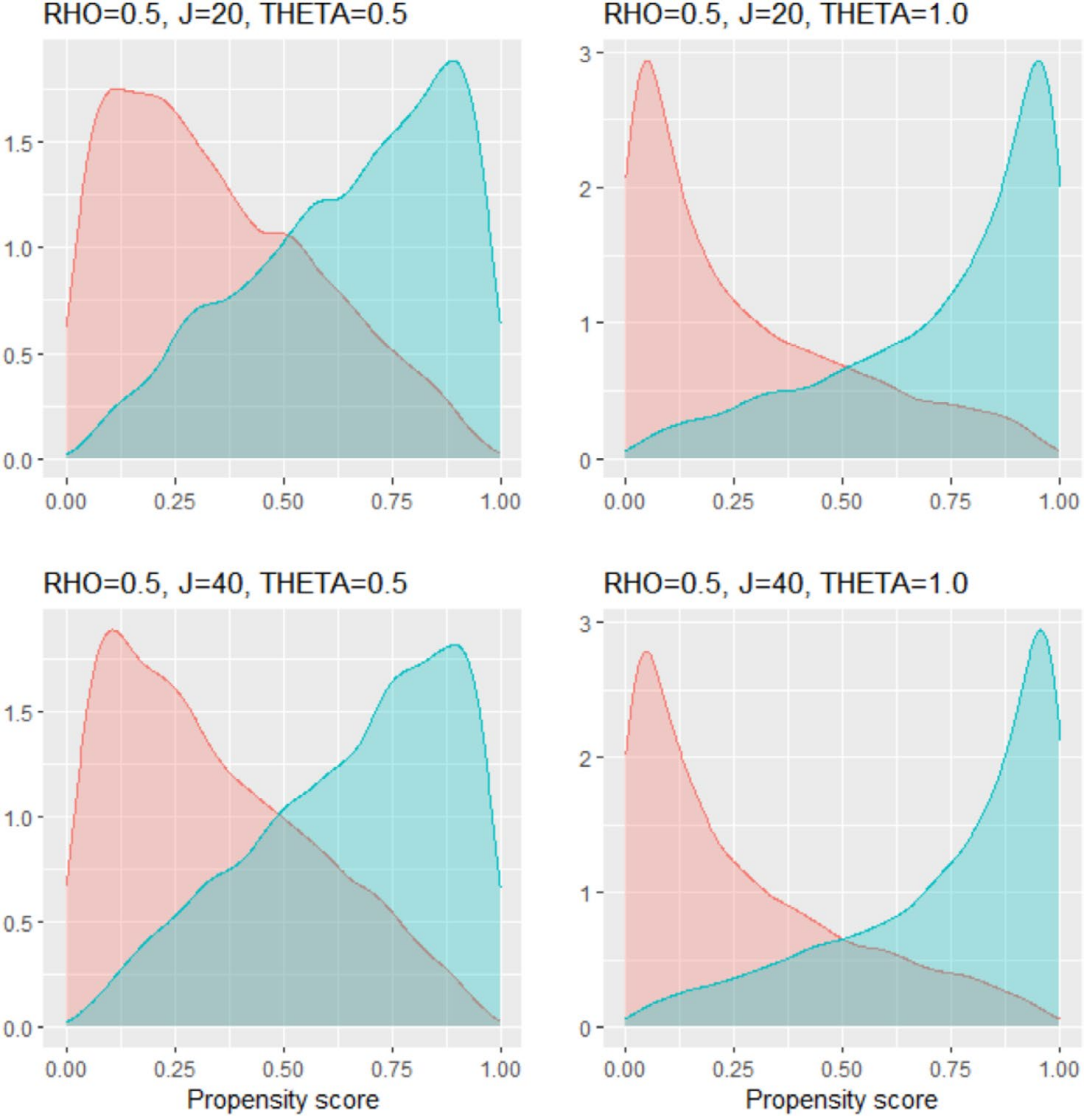
Distributions of the propensity scores across simulation scenarios that differ by the number of covariates (J) and instrument strength (*θ*). The correlation coefficient among the covariates (*ρ*) is fixed at 0.5. The red and blue density curves represent the propensity scores for the control and treatment groups, respectively.

To examine how the simulation parameters affected the PS distribution, using the simulated data sets with a sample size of 10 000, we graphed the PS distributions across simulation scenarios that differ by the correlation $\left(\rho \right)$ among the covariates, number of covariates $\left(J\right)$, and IV strength $\left(\theta \right)$. Figures 1 and 2 display those when ρ was 0 and 0.5, respectively, demonstrating that the PS overlap between groups diminishes with increasing ρ or θ. In Table 2, we calculated the percentages of the PSs outside the range [0.1, 0.9] across the simulation scenarios that differ by the three parameters. We can see that the percentages of extreme PSs were meaningfully affected by the relationship between the covariates and IV strength. Under our simulation models, the directions of the associations were the same among the covariates in the PS model. As a result, a large correlation between the covariates and strong IVs increased the number of extreme PSs. Accordingly, with 20 covariates, as the correlation coefficient increased from 0 to 0.5, the percentage of the PSs outside the range [0.1, 0.9] increased from 0.03 to 0.16 when the IV was moderate $\left(\theta =0.5\right)$, and from 0.16 to 0.37 when the IV was strong $\left(\theta =1\right)$. Similar percentages were observed with 40 covariates.

**TABLE 2:** Percentages of the propensity scores outside the range [0.1, 0.9] according to different simulation settings.

	Number of covariates $\left(J\right)$	20		40	
	IV strength $\left(\theta \right)$	Moderate (0.5)	Large (1.0)	Moderate (0.5)	Large (1.0)
Rho $\left(\rho \right)$	0	0.03	0.16	0.02	0.16
	0.5	0.16	0.37	0.17	0.36

Table 3 shows the simulation results for homogeneous treatment effects when the covariates were independent. If comparing “Target” and “Confounders”, we can see that including the stronger IVs in a PS model inflated MSE the more. The MSE was greatest when all the covariates were used for the PS models (“All covariates”), which means that including spurious variables decreased the accuracy of the AIPTW estimators. OAL performed better than “Confounders” when the IVs were strong. SBW outperformed OAL and SCS in terms of MSE. Particularly, SBW performed very well when the IV strength was moderate $\left(\theta =0.5\right)$: the R‐MSE of SBW was less than 1.20. OAL and SBW gave valid CovRates for all scenarios, while SCS yielded under‐CovRates when either the IVs were strong, or the number of covariates was 40.

**TABLE 3:** Simulation results with a sample size of 500 when there are no correlations among the covariates $\left(\rho =0\right)$ and treatment effects are homogeneous.

Number of	IV	Estimation	Performance measures					
Covariates	Strength	Methods	Bias	MSE	R‐MSE	EmpSE	AgeSE	CovRate
20	0.5	Target	0.007	0.035	1.000	0.188	0.190	0.964
		Confounders	0.008	0.044	1.259	0.211	0.207	0.962
		All covariates	0.008	0.046	1.306	0.214	0.212	0.952
		OAL	0.007	0.045	1.266	0.211	0.204	0.948
		SCS	0.004	0.044	1.247	0.210	0.201	0.946
		SBW	0.006	0.041	1.160	0.202	0.196	0.948
	1.0	Target	0.010	0.029	1.000	0.170	0.185	0.972
		Confounders	0.012	0.076	2.629	0.275	0.257	0.954
		All covariates	0.013	0.086	2.981	0.293	0.269	0.952
		OAL	0.010	0.052	1.806	0.228	0.224	0.940
		SCS	0.014	0.060	2.098	0.246	0.224	0.916
		SBW	0.014	0.042	1.449	0.204	0.214	0.966
40	0.5	Target	0.006	0.038	1.000	0.196	0.189	0.948
		Confounders	0.011	0.046	1.203	0.215	0.207	0.944
		All covariates	0.017	0.056	1.457	0.236	0.220	0.934
		OAL	0.017	0.052	1.352	0.227	0.208	0.930
		SCS	0.014	0.050	1.300	0.223	0.198	0.908
		SBW	0.012	0.045	1.180	0.213	0.195	0.922
	1.0	Target	0.003	0.035	1.000	0.186	0.186	0.946
		Confounders	0.011	0.075	2.163	0.273	0.257	0.946
		All covariates	0.006	0.096	2.777	0.310	0.283	0.944
		OAL	0.014	0.072	2.073	0.267	0.245	0.942
		SCS	0.011	0.064	1.852	0.253	0.216	0.910
		SBW	0.004	0.051	1.470	0.225	0.213	0.936

Abbreviations: AvgSE: average model‐based standard error; CovRate: 95% confidence interval coverage rate; EmpSE: empirical standard error; MSE: mean squared error; OAL: outcome‐adaptive lasso; R‐MSE: relative mean squared error; SBW: stable balancing weighting; SCS: stable confounder selection.

Table 4 shows the simulation results for constant treatment effects when the covariates were strongly correlated to each other. Surprisingly, SBW significantly outperformed both OAL, SCS, and even “Target” in terms of MSE for all scenarios. This superior performance of SBW over “Target” resulted from the fact that approximately 36% of the PSs were outside the range [0.1, 0.9]. Again, OAL performed better than “Confounders” in terms of MSE when the IVs were strong. OAL and SBW gave valid CovRates. SCS outperformed OAL in terms of MSE but yielded under‐coverage rates because its AvgSEs were substantially smaller than EmpSEs.

**TABLE 4:** Simulation results with a sample size of 500 when there are strong correlations among the covariates $\left(\rho =0.5\right)$ and treatment effects are homogeneous.

Number of	IV	Estimation	Performance measures					
Covariates	Strength	Methods	Bias	MSE	R‐MSE	EmpSE	AgeSE	CovRate
20	0.5	Target	0.006	0.063	1.000	0.250	0.237	0.942
		Confounders	0.010	0.077	1.236	0.278	0.255	0.930
		All covariates	0.015	0.091	1.460	0.302	0.270	0.928
		OAL	0.009	0.079	1.268	0.282	0.257	0.930
		SCS	0.014	0.074	1.175	0.271	0.229	0.900
		SBW	0.009	0.050	0.795	0.223	0.216	0.936
	1.0	Target	0.015	0.080	1.000	0.283	0.261	0.936
		Confounders	0.020	0.306	3.821	0.553	0.374	0.934
		All covariates	0.025	0.385	4.801	0.620	0.410	0.930
		OAL	0.001	0.193	2.411	0.440	0.354	0.920
		SCS	0.005	0.116	1.447	0.341	0.254	0.854
		SBW	0.005	0.067	0.835	0.259	0.244	0.930
40	0.5	Target	0.012	0.059	1.000	0.242	0.232	0.938
		Confounders	0.018	0.084	1.424	0.289	0.261	0.948
		All covariates	0.010	0.118	2.008	0.344	0.291	0.934
		OAL	0.004	0.093	1.592	0.306	0.270	0.928
		SCS	0.012	0.073	1.238	0.270	0.220	0.880
		SBW	0.017	0.050	0.858	0.224	0.214	0.930
	1.0	Target	0.002	0.067	1.000	0.259	0.255	0.940
		Confounders	0.003	0.239	3.575	0.490	0.369	0.942
		All covariates	0.011	0.342	5.118	0.586	0.439	0.956
		OAL	0.001	0.230	3.436	0.480	0.392	0.952
		SCS	0.006	0.113	1.682	0.336	0.235	0.828
		SBW	0.001	0.056	0.834	0.236	0.244	0.972

Abbreviations: AvgSE: average model‐based standard error; CovRate: 95% confidence interval coverage rate; EmpSE: empirical standard error; MSE: mean squared error; OAL: outcome‐adaptive lasso; R‐MSE: relative mean squared error; SBW: stable balancing weighting; SCS: stable confounder selection.

Tables 5 and 6 present the simulation results with heterogeneous treatment effects when the correlation coefficient among the covariates was 0 and 0.5, respectively. The results with heterogeneous treatment effects were similar to those with homogeneous treatment effects. SBW outperformed OAL and SCS when there were no correlations among the covariates. Notably, while both methods produced correct CovRates, SBW outperformed “Target” by achieving smaller EmpSE and MSE when covariates were correlated $\left(\rho =0.5\right)$. The R‐MSE of SBW ranged from 0.588 to 0.632, demonstrating its superior performance under these conditions.

**TABLE 5:** Simulation results with a sample size of 500 when there are no correlations among the covariates $\left(\rho =0\right)$ and treatment effects are heterogeneous.

Number of	IV	Estimation	Performance measures					
Covariates	Strength	Methods	Bias	MSE	R‐MSE	EmpSE	AgeSE	CovRate
20	0.5	Target	0.008	0.041	1.000	0.201	0.207	0.960
		Confounders	0.008	0.056	1.391	0.238	0.229	0.954
		All covariates	0.006	0.056	1.385	0.237	0.232	0.948
		OAL	0.007	0.054	1.324	0.232	0.222	0.948
		SCS	0.003	0.050	1.242	0.225	0.215	0.946
		SBW	0.007	0.045	1.121	0.213	0.209	0.958
	1.0	Target	0.010	0.034	1.000	0.185	0.200	0.968
		Confounders	0.009	0.078	2.263	0.279	0.275	0.946
		All covariates	0.008	0.089	2.595	0.299	0.288	0.946
		OAL	0.007	0.062	1.819	0.250	0.248	0.930
		SCS	0.009	0.069	2.010	0.263	0.237	0.922
		SBW	0.014	0.047	1.371	0.217	0.227	0.948
40	0.5	Target	0.011	0.045	1.000	0.212	0.206	0.952
		Confounders	0.018	0.056	1.243	0.236	0.227	0.938
		All covariates	0.023	0.070	1.565	0.264	0.243	0.934
		OAL	0.023	0.062	1.384	0.249	0.228	0.940
		SCS	0.020	0.059	1.321	0.243	0.213	0.916
		SBW	0.018	0.052	1.147	0.227	0.208	0.950
	1.0	Target	0.010	0.037	1.000	0.193	0.201	0.946
		Confounders	0.001	0.090	2.414	0.301	0.283	0.954
		All covariates	0.002	0.118	3.160	0.344	0.312	0.948
		OAL	0.001	0.085	2.279	0.292	0.268	0.938
		SCS	0.006	0.073	1.939	0.270	0.231	0.906
		SBW	0.002	0.053	1.414	0.230	0.225	0.944

Abbreviations: AvgSE: average model‐based standard error; CovRate: 95% confidence interval coverage rate; EmpSE: empirical standard error; MSE: mean squared error; OAL: outcome‐adaptive lasso; R‐MSE: relative mean squared error; SBW: stable balancing weighting; SCS: stable confounder selection.

**TABLE 6:** Simulation results with a sample size of 500 when there are strong correlations among the covariates $\left(\rho =0.5\right)$ and treatment effects are heterogeneous.

Number of	IV	Estimation	Performance measures					
Covariates	Strength	Methods	Bias	MSE	R‐MSE	EmpSE	AgeSE	CovRate
20	0.5	Target	0.011	0.090	1.000	0.300	0.294	0.962
		Confounders	0.023	0.119	1.327	0.345	0.318	0.952
		All covariates	0.038	0.146	1.624	0.380	0.332	0.952
		OAL	0.025	0.111	1.232	0.332	0.316	0.954
		SCS	0.019	0.086	0.961	0.294	0.255	0.916
		SBW	0.008	0.055	0.617	0.236	0.237	0.946
	1.0	Target	0.022	0.116	1.000	0.340	0.323	0.948
		Confounders	0.003	0.348	3.009	0.590	0.453	0.940
		All covariates	0.011	0.505	4.372	0.711	0.492	0.940
		OAL	0.002	0.351	3.038	0.593	0.435	0.936
		SCS	0.008	0.149	1.286	0.386	0.284	0.852
		SBW	0.008	0.073	0.632	0.270	0.268	0.938
40	0.5	Target	0.004	0.094	1.000	0.306	0.294	0.950
		Confounders	0.001	0.150	1.595	0.387	0.333	0.954
		All covariates	0.002	0.192	2.045	0.438	0.370	0.952
		OAL	0.001	0.146	1.559	0.383	0.339	0.948
		SCS	0.005	0.095	1.013	0.308	0.247	0.886
		SBW	0.011	0.055	0.588	0.235	0.236	0.950
	1.0	Target	0.015	0.128	1.000	0.357	0.333	0.952
		Confounders	0.005	0.473	3.705	0.689	0.479	0.952
		All covariates	0.002	0.712	5.573	0.845	0.575	0.964
		OAL	0.010	0.500	3.917	0.708	0.509	0.960
		SCS	0.004	0.140	1.093	0.374	0.266	0.836
		SBW	0.003	0.063	0.495	0.252	0.267	0.974

Abbreviations: AvgSE: average model‐based standard error; CovRate: 95% confidence interval coverage rate; EmpSE: empirical standard error; MSE: mean squared error; OAL: outcome‐adaptive lasso; R‐MSE: relative mean squared error; SBW: stable balancing weighting; SCS: stable confounder selection.

**FIGURE 3:**
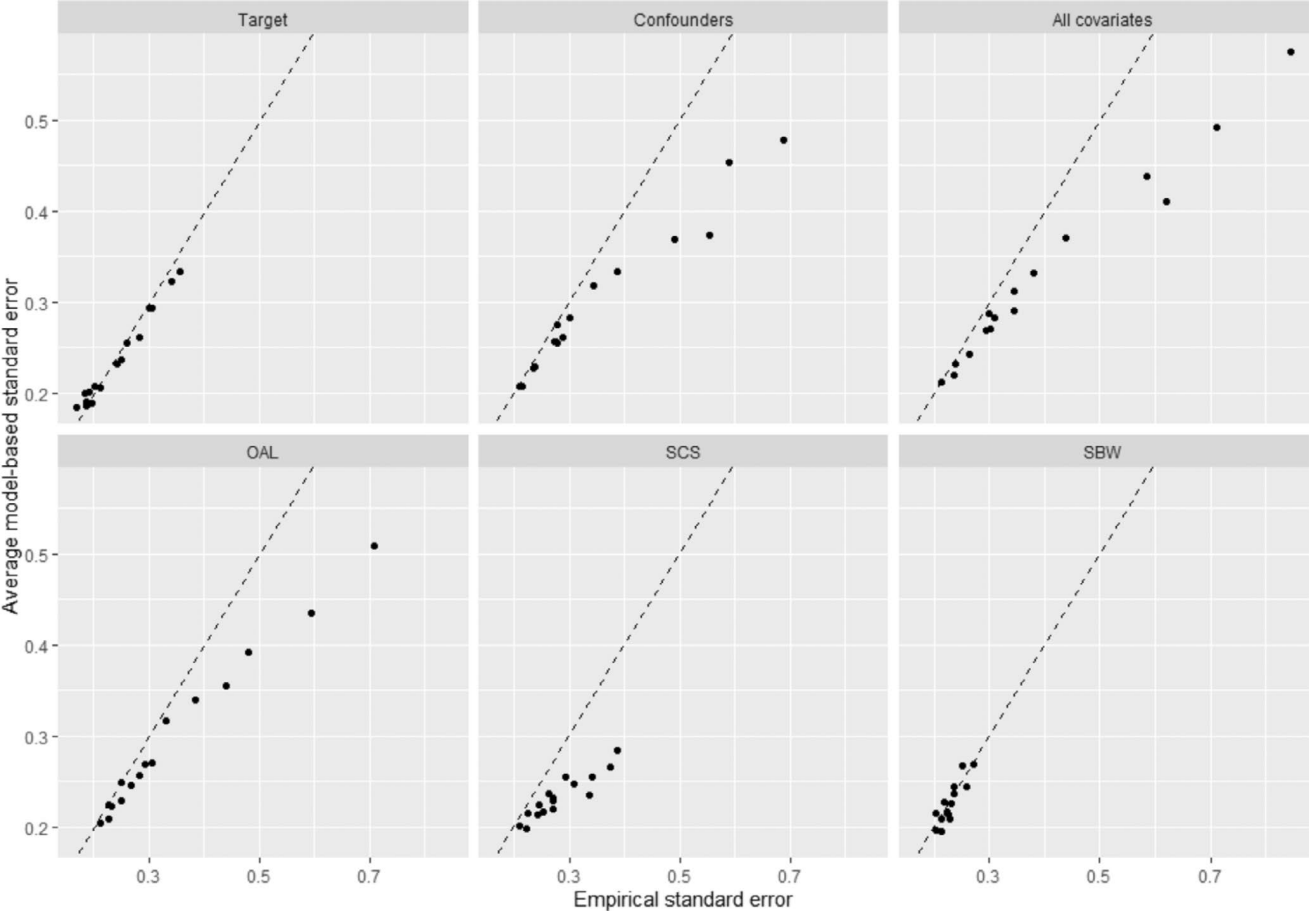
A comparison of average model‐based standard errors (SEs) versus empirical standard errors for a sample size of 500. The dotted line represents when the estimated SEs equal the empirical SEs. OAL, outcome‐adaptive lasso; SBW, stable balancing weighting; SCS, stable confounder selection.

**FIGURE 4:**
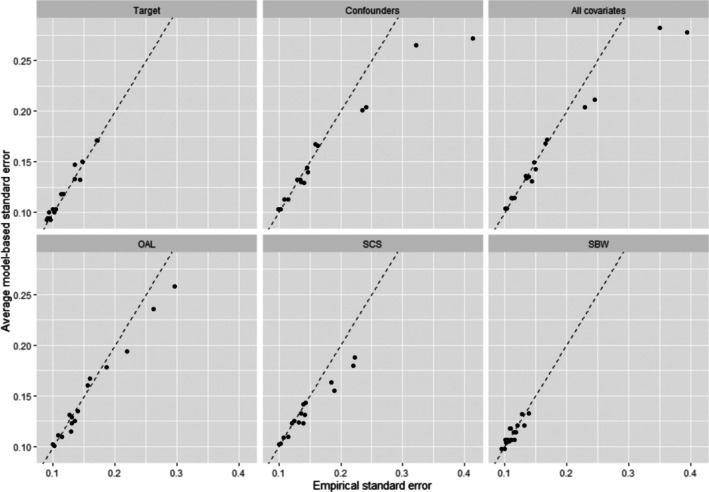
A comparison of average model‐based standard errors (SEs) versus empirical standard errors for a sample size of 2000. The dotted line represents when the estimated SEs equal the empirical SEs. OAL: outcome‐adaptive lasso; SBW: stable balancing weighting; SCS: Stable confounder selection.

Figures 3 and 4 compare the average model‐based standard errors (SEs) with the empirical SEs obtained from all six methods across all simulation scenarios, for sample sizes of 500 and 2000, respectively. Both Target and SBW produced SE estimates that closely matched their empirical counterparts across all scenarios. Notably, the estimated and empirical SEs of SBW were smaller than those of Target in the presence of strong IVs correlated with other covariates. In contrast, OAL and SCS produced inaccurate SE estimates in scenarios with relatively large empirical SEs for n=500, particularly when IVs were correlated with other covariates. However, the accuracy of SE estimates from OAL and SCE improved substantially as the sample size increased to 2000.

Tables S1–S4 show the results when the sample size was increased from 500 to 2000. The main results were the same as those with n=500: SBW outperformed OLA, SCS, and even “Target” when the covariates were correlated to each other.

## 4 Application to Abciximab Data

Abciximab is an adjunctive pharmacotherapy during initial PCI to reduce the risk of periprocedural ischemic events. Kereiakes et al. [32] examined the effectiveness of abciximab on the patients who received PCI at The Christ Hospital in Cincinnati, Ohio. They found that abciximab was associated with a survival benefit to 6 months after PCI. We reanalyzed the data from this observational study of 996 PCI patients to address a causal question of whether receiving abciximab reduces 6‐month mortality. In this data, abciximab was given to 698 patients, and the remaining 298 patients received PCI alone. For meaningful interpretations of the PS models, we centered height by 170 cm and scaled it by 10 cm. We also centered LVEF by 50 percentage points and scaled it by 10 percentage points.

We adopted the causal roadmap [22] to address the causal question regarding abciximab. The causal roadmap is particularly useful to clearly distinguish knowledge‐based assumptions (or causal assumptions) from convenience‐based assumptions. We began by specifying causal knowledge based on a directly acyclic graph (DAG) as depicted in Figure 3. In the DAG, the demographic and procedural characteristics $\left(X\right)$, measured by the seven pretreatment variables, and abciximab $\left(Z\right)$ affect 6‐month mortality $\left(Y\right)$, but the unmeasured factors that determine the nodes could be correlated to each other. The causal knowledge encoded in the DAG can be represented by the following causal structural equation

$$X=f_{X}\left(U_{X}\right),Z=f_{Z}\left(X,U_{Z}\right),\text{and}\;Y=f_{Y}\left(X,Z,U_{Y}\right),$$

where $f_{X}$, $f_{Z}$, and $f_{Y}$ are the deterministic functions of their respective parents and the unmeasured factors $U=\left(U_{X},U_{Z},U_{Y}\right)$ with no restriction on the distribution of U.

**FIGURE 5:**
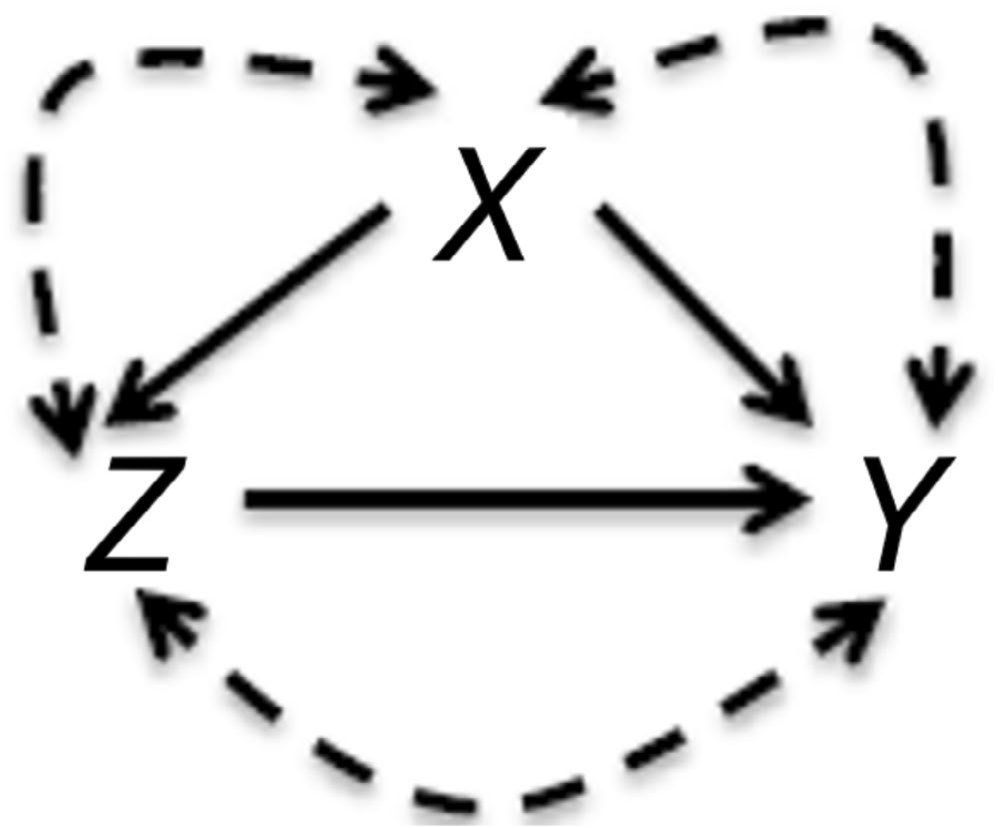
Directed acyclic graph representing the data generating process of the abciximab data. X, Z, and Y represent measured covariates, abciximab, and 6‐month mortality. The dashed arrows represent that the unmeasured factors that determine the nodes could be correlated to each other.

**FIGURE 6:**
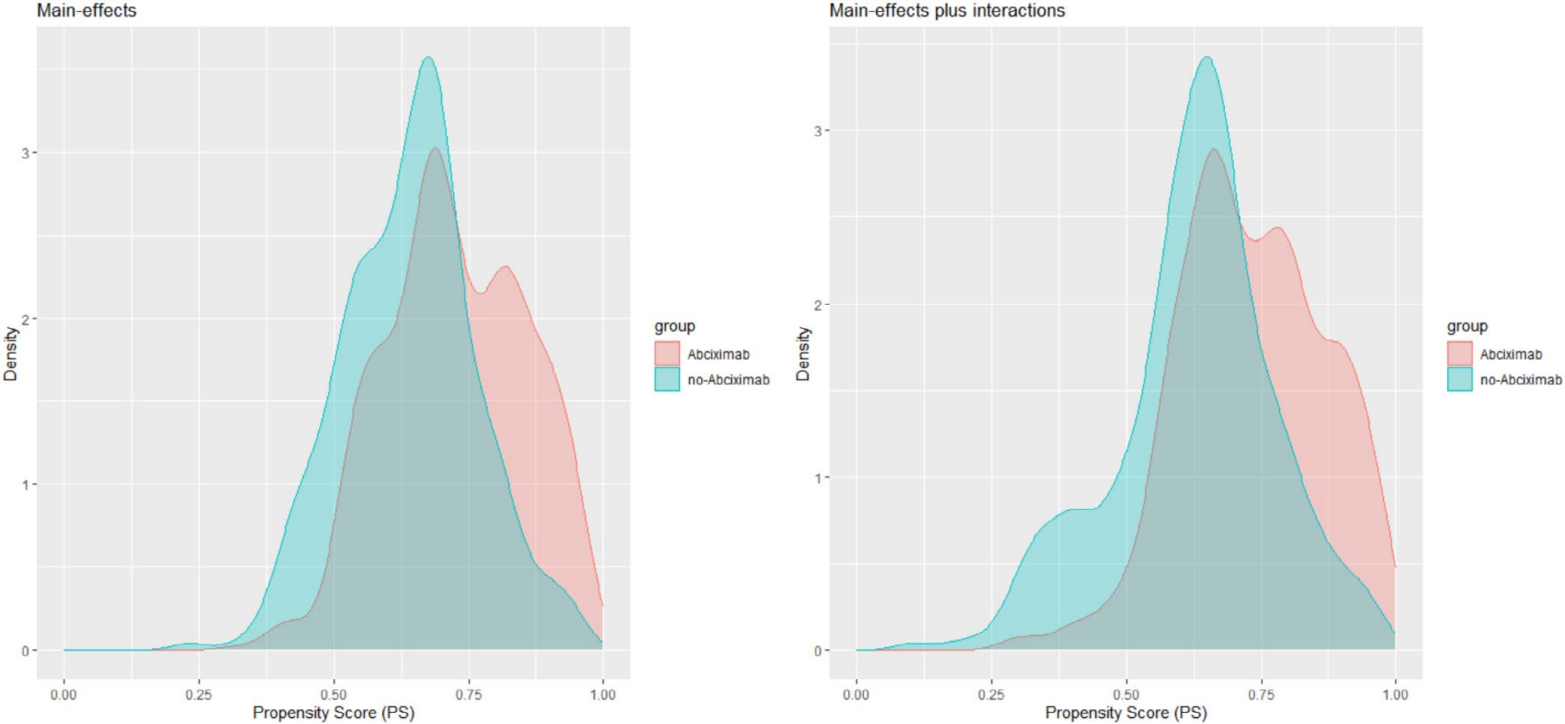
Propensity score (PS) densities for the abciximab and no‐abciximab groups. The left and right panels show the densities when the PS models are adjusted for the main effects of the covariates and those plus the squared and interaction terms.

We assumed the observed data were independent random draws from the causal model in Figure 3 and selected our target estimand as the ATE of receiving abciximab on 6‐month mortality. In the counterfactual framework, this ATE is defined as the difference in 6‐month mortality rates that would have been observed if all patients had received versus not received abciximab. Under the causal model (Figure 5), the effect of abciximab on 6‐month mortality was unidentified due to the unmeasured factors. Thus, we imposed a convenience‐based assumption that the extended covariate set, including all two‐way interactions and squared terms of all continuous covariates, indirectly can address the unmeasured factors. Accordingly, we identified the ATE because exchangeability should hold under the convenience‐based assumption; however, including the interactions based on procedural characteristics such as stent and AMI induced positivity violations. To circumvent this issue, we considered the interactions based on only patient and clinical demographics (female gender and DMD), which led to a total of 21 control variables (Table 7). Figure 6 shows the PS densities when the PS models were adjusted for the main effects only and those plus the interaction and squared terms. Adding the extended variables to the model made the limited overlap more severe.

**TABLE 7:** Regression coefficients of the PS models. GLM, OAL, and SCS indicate the traditional logistic model, outcome‐adaptive lasso model, and logistic model using the covariates selected by the stable confounder selection method, respectively. The covariates are ordered by SCS.

Covariates	GLM	OAL	SCS
Ves1proc	0.41	0.27	0.91
Acutemi	1.38	0.53	1.54
Ejecfrac	−0.17	−0.18	−0.15
Stent	0.34	0.16	0.36
Diabetic:ves1proc	−0.42	−0.27	−0.27
Stent:diabetic	1.03	0.36	−0.53
Height:female	−0.36	−0.20	0.90
Female	−0.73	−0.34	−0.36
Diabetic:acutemi	−0.87	−0.14	−0.92
Stent:female	−0.07	−0.03	−0.06
Female:diabetic	−0.54	−0.14	−0.34
Ejecfrac^2^	−0.02	−0.07	0.00
Height^2^	−0.03	−0.08	0.00
Ves1proc^2^	0.10	0.27	0.00
Female:ves1proc	0.34	0.23	0.00
Height:diabetic	−0.13	−0.07	0.00
Diabetic	−0.18	−0.10	0.00
Female:acutemi	0.36	0.00	0.00
Female:ejecfrac	−0.10	0.00	0.00
Diabetic:ejecfrac	0.10	0.00	0.00
Height	−0.04	−0.04	0.00

Abbreviations: acutemi, acute myocardial infarction; diabetic, diabetes mellitus diagnosis; ejecfrac, left ventricular ejection fraction; stent, coronary stent deployment; ves1proc, number of vessels involved in the patient's initial percutaneous coronary intervention.

We used the traditional AIPTW estimator and those based on OAL, SCS, and SBW to estimate the ATE of abciximab. The outcome model for 6‐month mortality and the PS model were fitted using logistic regression models using the covariates listed in Table 7. The SBW weights were obtained by balancing the covariates in Table 7 toward their overall means. We reported the point estimates, their SEs, and lower and upper bounds of 95% confidence intervals in Table 8. The point estimates were similar to each other, implying that receiving abciximab reduced 6‐month mortality by 5.5 (AIPTW and OAL) or 5.2 (SCS and SBW) percentage points. The SEs of SCS and SBW were 1.6 and 1.7 percentage points, respectively, while those of AIPTW and OAL were the same at 2.1 percentage points.

**TABLE 8:** Average treatment effects of receiving abciximab on 6‐month mortality. Point estimates, their standard errors (SEs), and upper (LB) and lower (UB) bounds of 95% confidence intervals. Positive values indicate reduced mortality by abciximab.

Method	Estimate (%)	SE (%)	LB (%)	UB (%)
GLM	5.5	2.1	1.3	9.6
OAL	5.5	2.1	1.4	9.6
SCS	5.2	1.6	2.1	8.3
SBW	5.2	1.7	1.7	8.6

Abbreviations: GLM, logistic regression; OAL, outcome‐adaptive lasso; SBW, stable balancing weighting; SCS, stable confounder selection.

We presented the regression coefficients of the traditional logistic PS model, the OAL model, and the logistic model using the covariates selected by SCS in Table 7. We ordered the covariates based on their associations with abciximab and 6‐month mortality, following the approach proposed by Loh and Vansteelandt. Notably, LVEF, NVIP, stent deployment, and AMI, assumed to be strong confounders, were consistently selected by OAL and identified as the most important covariates by SCS.

The effects of using OAL and SCS to reduce the influence of potential IVs or near‐IVs on the PSs are evident. For example, the DMD × NVIP interaction had an odds ratio of 0.66 in the logistic PS model, but its effect size was reduced to 0.76 in both the OAL and SCS models. Similarly, the Female × DMD had an odds ratio of 0.58, but its effect size was reduced to 0.87 and 0.71 in the OAL and SCS models, respectively. The Female × NVIP interaction, which had an odds ratio of 1.4 in the logistic PS model, was reduced to 1.26 by OAL and excluded from the adjustment set by SCS. Finally, the Female × AMI interaction, with an odds ratio of 1.43 in the logistic PS model, had its coefficient shrunken exactly toward 0 by OAL and excluded from the adjustment set by SCS.

**FIGURE 7:**
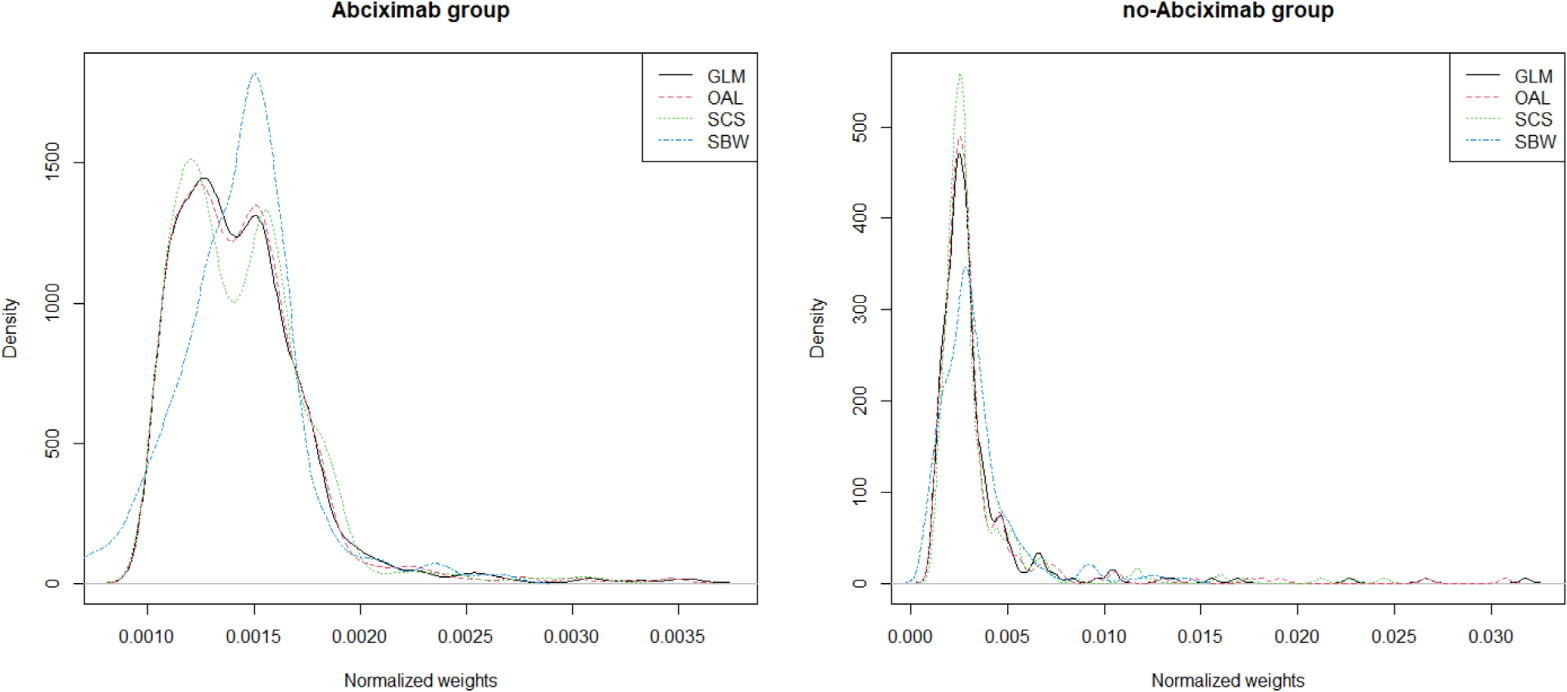
Kernel densities of normalized weights in the abciximab and no‐abciximab groups. GLM, logistic regression; OAL, outcome‐adaptive lasso; SBW, stable balancing weighting; SCS, stable confounder selection.

We evaluated several diagnostic measures, including effective sample size (ESS), maximum weight, weight distribution, and population standardized difference (PSD), to gain deeper insights into the performance and limitations of each method. The ESS reflects the amount of information the weighted sample provides about the target population [33, 34]. The ESS for group z is defined as ${\left\{\sum_{i=1}^{n}I\left(Z_{i}=z\right){\hat{W}}_{i}\left(X_{i}\right)\right\}}^{2}/\left\{\sum_{i=1}^{n}1\left(Z_{i}=z\right){\hat{W}}_{i}^{2}\left(X_{i}\right)\right\}$ and maximized when all weights are the same, indicating no loss in precision due to weighting. It is particularly sensitive to the presence of extreme weights. Table 9 reports the ESS and maximum weight for each group, alongside the corresponding values from the crude weights for comparison, defined as $1/\sum_{i=1}^{n}I\left(Z_{i}=z\right)$ for group z. SBW achieved the highest ESS and the lowest maximum weight, especially in the no‐abciximab group. Furthermore, as shown in Figure 7, SBW yielded the most concentrated (least variable) distributions of normalized weights.

**TABLE 9:** Effective sample sizes and maximum weights. Crude: unweighted; GLM: logistic regression; OAL: outcome‐adaptive lasso; SBW: stable balancing weighting; SCS: stable confounder selection.

	Effective sample size		Maximum weight × 100	
	Abciximab	No‐abciximab	Abciximab	No‐abciximab
Crude	698	298	0.14	0.34
GLM	665	159	0.36	3.18
OAL	666	161	0.35	3.07
SCS	668	172	0.31	2.44
SBW	669	211	0.27	1.44

**FIGURE 8:**
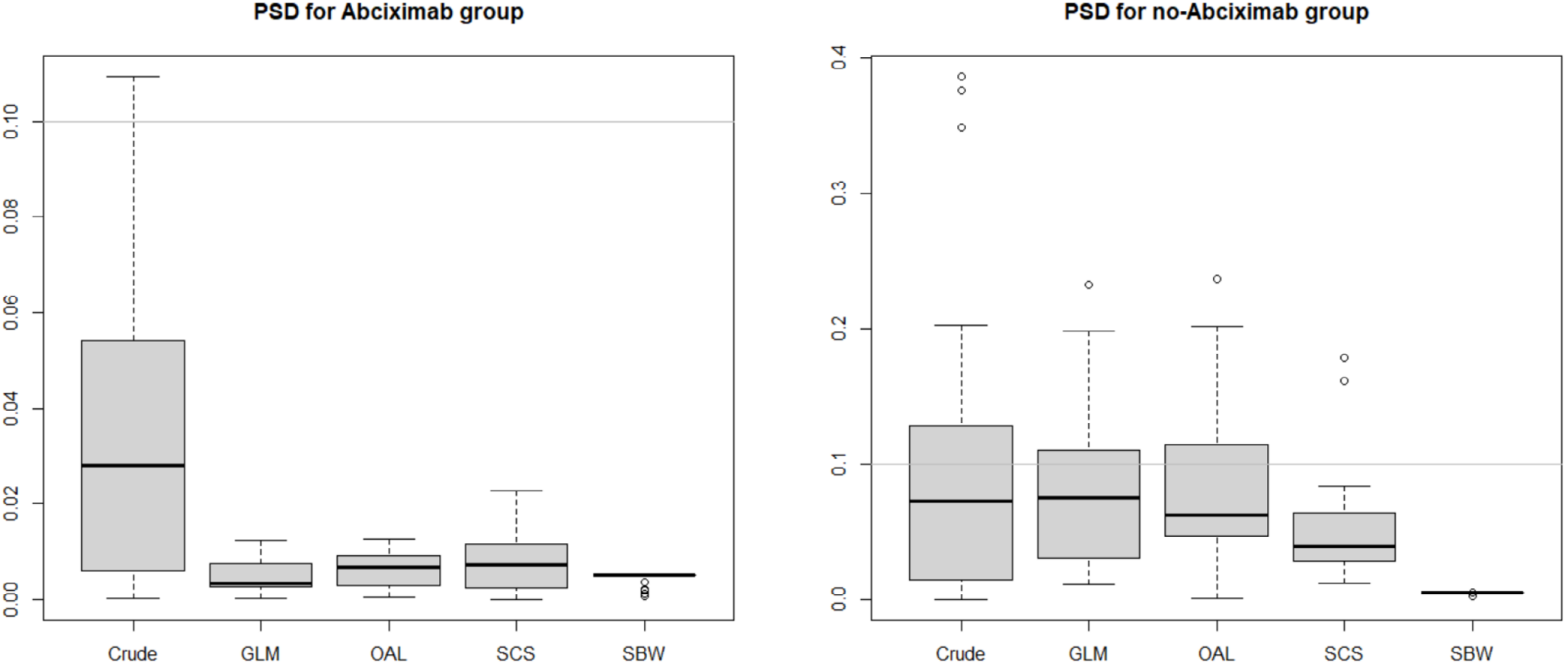
Boxplots for the population standardized difference (PSD) for normalized weights in the abciximab and no‐abciximab groups. The gray horizontal line indicates adequate balance at 0.1. Crude: unweighted; GLM, logistic regression; OAL, outcome‐adaptive lasso; SBW, stable balancing weighting; SCS, stable confounder selection.

We also evaluated PSD to examine how well the weights balanced the groups toward the target population. We defined ${\bar{X}}_{j}\left(z\right)=\sum_{i=1}^{n}X_{\mathrm{ij}}1\left(Z_{i}=z\right){\hat{W}}_{i}\left(X_{i}\right)/\sum_{i=1}^{n}1\left(Z_{i}=z\right){\hat{W}}_{i}\left(X_{i}\right)$ as the weighted mean of covariate $X_{\mathrm{ij}}$ and $S_{j}^{2}\left(z\right)$ as the unweighted variance of $X_{\mathrm{ij}}$ from group z. Further, we defined ${\bar{X}}_{j}=\sum_{i=1}^{n}X_{\mathrm{ij}}/n$ as the mean of $X_{\mathrm{ij}}$ in the whole sample. The PSD for each covariate $X_{\mathrm{ij}}$ for group z was defined as $\mathrm{PS}D_{j}\left(z\right)=\left|{\bar{X}}_{j}\left(z\right)-{\bar{X}}_{j}\right|/S_{j}\left(z\right)$. Figure 8 demonstrates that SBW produced PSD much smaller than the other methods did, particularly for the no‐abciximab group, for which all methods produced PSD values greater than 0.1.

## 5 Discussion

Addressing extreme PSs or practical or actual violations of positivity is essential to obtaining reliable IPTW estimators for the ATE in many circumstances. OAL, SCS, and SBW are promising methods to address extreme PSs, but they have different statistical properties. Through simulations, we demonstrated that SBW generally outperformed both OAL and SCS, yielding estimators with less bias and smaller MSEs, mainly when the degrees of IV strength and the correlations were large. In such settings, SBW even outperformed the benchmark method (“Target”) that used the true confounders and outcome predictors. Even though “Target” used the optimal covariate set, it relies on a modeling approach that maximizes the fit of a logistic regression model to produce PSs with the highest predictive accuracy. In contrast, SBW is a balancing approach that directly estimates weights without modeling, aiming to approximately balance covariates while minimizing weight dispersion. Our findings suggest that such a balancing approach can outperform the modeling approach, even when the latter uses a priori knowledge on the optimal covariate set. Our real example also demonstrated that SBW can accommodate a high‐dimensional set of covariates without losing precision.

Several methods have been proposed to address the issue of limited overlap in PS distributions. For instance, Crump et al. [35] introduced a rule‐of‐thumb for PS trimming, recommending the exclusion of subjects whose PSs fall outside the interval [0.1, 0.9]. Another strategy to reduce the influence of extreme PSs involves the use of tilting functions, which shift the focus toward subpopulations that deviate from the original sample but offer desirable statistical properties [36, 37]. Examples include overlap weights (OW), matching weights (MW), and entropy weights (EW), all of which continuously down‐weigh subjects in the tails of the PS distribution and emphasize subpopulations with strong treatment equipoise [4, 36, 38, 39]. While approaches such as PS trimming and OW are effective in managing extreme PSs, they inherently exclude some individuals or redefine the target population, which may introduce bias or limit generalizability [37]. In contrast, the methods compared in this study retain the full analytic sample and preserve the original population definition by appropriately adjusting weights or selecting covariates, thereby maintaining statistical precision and reducing the risk of unintended bias.

Another notable method is the incremental propensity score (IPS) [40], a relatively new method that circumvents positivity issues. IPS considers an alternative causal effect from shifting the PSs by a fixed amount rather than contrasting the counterfactual outcomes. IPS is useful to address causal questions in situations where it is unrealistic to force all population units to receive political or behavioral interventions [41, 42]. However, in a pharmacoepidemiology setting like our example, it is more natural to contrast the potential outcomes that would be observed if all patients received or did not receive a drug rather than to consider the causal effect of shifting their probabilities of receiving the drug.

Our simulations varied in IV strength, correlation structure, and the number of covariates. In particular, varying IV strength, especially including strong IVs, reflected a common challenge in epidemiological studies, where some variables are strongly associated with treatment but weakly or not at all associated with the outcome. Including such IVs can degrade the precision of effect estimates [7, 8, 9]. To assess how covariate correlation influences this issue, we examined both independent and moderately correlated covariates. We found that the presence of correlation, especially between IVs and other covariates, substantially reduced the performance of methods like OAL and SCS, which depend on variable selection through penalization or stability‐based criteria. This finding has important implications for applications to large‐scale data sources such as electronic health records and claims data, where covariates tend to be moderately to highly correlated as their number increases.

In our application, we assumed a simple causal model, which may be insufficient to represent a true data‐generating process. Alternatively, causal discovery algorithms can be used to find causal graphical models in data‐adaptive ways while accounting for prior causal knowledge. We refer interested readers to the introductory paper by Malinsky and Danks [43] and the references therein.

In this study, we considered single time‐point interventions. However, settings with multiple time‐point interventions are also common in the real world and more difficult to analyze due to potential time‐dependent confounding and informative censoring [44]. Moreover, simulating such longitudinal data to evaluate relevant statistical methods is challenging and would require a systematic data‐generating approach. The R package **simcausal** [45] is one of the tools to address this challenge. This package generates data based on nonparametric structural equation models, which allows users to consider complex causal relationships among variables. Developing and evaluating causal inference methods for time‐varying interventions using such a program tool as **simcausal** merit the future study.

We used AIPTW as our working effect estimator. AIPTW is doubly robust, meaning it provides two opportunities for consistent estimation of ATE: consistency is achieved if either the PS model or the outcome regression model is correctly specified. Moreover, when both models are correctly specified, AIPTW is the most efficient among the PS‐weighted estimators [28]. However, despite its robustness and efficiency under ideal conditions, doubly robust estimators like AIPTW can perform unreliably in settings with limited overlap [4, 6, 46]. Our simulations confirmed this limitation: under limited overlap induced by strong IVs, the model‐based SEs from AIPTW deviated substantially from their empirical counterparts, even when the PS model was correctly specified and priori knowledge about potential confounders was incorporated. Our findings show that SBW improved the performance of the AIPTW estimator by increasing precision and yielding more accurate SE estimates. In contrast, OAL and SCS produced inaccurate SE estimates in scenarios where strong IVs were correlated with other covariates. Since both methods involve variable selection, accounting for the additional variability introduced by the model selection process may be essential for obtaining accurate SE estimates.

The choice of method significantly influences study design. Traditional PS analysis emphasizes a clear separation between design and analysis phases: estimating the PS without using outcome information. In contrast, OAL and SCS incorporate outcome information directly, either by weighting the penalty term or prioritizing covariates, thereby requiring observed outcomes during estimation. SBW, however, aligns more closely with the design separation principle, as it estimates balancing weights independently of outcome data.

The choice of method also affects data collection strategies. OAL, which relies on penalized regression, is particularly well‐suited for settings with high‐dimensional covariates. Similarly, SBW can accommodate high‐dimensional data, as Wang and Zubizarreta [17] showed that the loss due to balancing a large number of covariates is bounded. In contrast, SCS may be less efficient in high‐dimensional settings, as it evaluates the stability of treatment effect estimates sequentially, resulting in computational costs that increase linearly with the number of covariates.

Traditional approaches for generalizing treatment effect estimates to a target population construct weights by separately modeling the PS and the study selection probability, then multiplying inverses of their estimates [47, 48]. Applying OAL and SCS to generalizability in this context is not straightforward, as incorporating the study selection model into the penalization or stability‐based selection frameworks presents methodological challenges. However, SBW provides a more direct and robust approach to improving generalizability by weighing the study sample to match the covariate distribution of a specific target population [49]. Notably, SBW circumvents the need to estimate study selection and treatment assignment probabilities separately. More importantly, it requires only summary statistics, rather than individual‐level data, from the target population, thereby enhancing its practical applicability.

The way methods select covariates and estimate PS weights significantly impacts the interpretability of effect estimates and the definition of the target population. OAL prioritizes the selection of true confounders and predictors of the outcome, while excluding covariates associated only with treatment. As a result, the definition of the ATE is more influenced by the confounders and outcome predictors than IVs, enabling efficient estimation. SBW, on the other hand, explicitly balances all available covariates to resemble the target population. This approach yields the ATE that reflects the full covariate set. SCS screens out covariates that do not contribute to confounding control and selects a set that produces an effect estimate the least sensitive to the adjustment set. Consequently, SCS focuses on identifying and adjusting for true confounders. This framework enhances the interpretability of the target estimand by selecting a minimal sufficient adjustment set.

Our simulations focused on evaluating how well the methods (OAL, SCS, and SBW) handle IVs in weighting, under the assumptions of no unmeasured confounding and correct specification of the outcome and PS models. However, these assumptions may not be held in real‐world settings. Therefore, future research should investigate the performance of these methods under limited overlap, unmeasured confounding, and model misspecification, to better understand their robustness in practical applications.

### 5.1 Plain Language Summary

Propensity score (PS) weighting is a popular method to estimate treatment effects. A PS represents the probability that a particular subject receives treatment given their observed characteristics, also called covariates. Among those covariates, some variables, known as instrumental variables (IVs), could be associated with only treatment but not with the outcome. Many studies showed that IVs could make PS weighting unreliable. This study compares three promising statistical methods to mitigate the impact of IVs: the outcome‐adaptive lasso (OAL), stable balancing weighting (SBW), and stable confounder selection (SCS). Our simulation results demonstrate that SBW generally outperforms OAL and SCS notably when IVs are strongly associated with treatment.

## Author Contributions

Byeong Yeob Choi conceived the research question, conducted analyses, and wrote the first draft of the manuscript. M. Alan Brookhart provided analytic advice and critical interpretation of findings. All authors edited and approved the final manuscript.

## Ethics Statement

The authors have nothing to report.

## Conflicts of Interest

The authors declare no conflicts of interest.

## Supporting information


**Data S1.** Supporting Information.

**Data S2.** Supporting Information.

**Data S3.** Supporting Information.

## References

[1] P. R. Rosenbaum and D. B. Rubin , “The Central Role of the Propensity Score in Observational Studies for Causal Effects,” Biometrika 70 (1983): 41–55.

[2] J. K. Lunceford and M. Davidian , “Stratification and Weighting via the Propensity Score in Estimation of Causal Treatment Effects: A Comparative Study,” Statistics in Medicine 23 (2004): 2937–2960. 15351954 10.1002/sim.1903

[3] B. Y. Choi , “Subclassification Estimation of the Weighted Average Treatment Effect,” Biometrical Journal 63 (2021): 1706–1728. 34270815 10.1002/bimj.202000310

[4] Y. Zhou , R. A. Matsouaka , and L. Thomas , “Propensity Score Weighting Under Limited Overlap and Model Misspecification,” Statistical Methods in Medical Research 29 (2020): 3721–3756. 32693715 10.1177/0962280220940334

[5] D. E. Ho , K. Imai , G. King , and E. A. Stuart , “Matching as Nonparametric Preprocessing for Reducing Model Dependence in Parametric Causal Inference,” Political Analysis 15 (2007): 199–236.

[6] J. D. Y. Kang and J. L. Schafer , “Demystifying Double Robustness: A Comparison of Alternative Strategies for Estimating a Population Mean From Incomplete Data,” Statistical Science 22 (2007): 523–539, https://projecteuclid.org/journals/statistical‐science/volume‐22/issue‐4/Demystifying‐Double‐Robustness‐‐A‐Comparison‐of‐Alternative‐Strategies‐for/10.1214/07‐STS227.full. 10.1214/07-STS227 PMC2397555 18516239

[7] M. A. Brookhart , S. Schneeweiss , K. J. Rothman , R. J. Glynn , J. Avorn , and T. Stürmer , “Variable Selection for Propensity Score Models,” American Journal of Epidemiology 163 (2006): 1149–1156. 16624967 10.1093/aje/kwj149 PMC1513192

[8] B. Y. Choi and M. A. Brookhart , “Effects of Adjusting for Instrumental Variables on the Bias and Precision of Propensity Score Weighted Estimators: Analysis Under Complete, Near, and No Positivity Violations,” Clinical Epidemiology 15 (2023): 1055–1068. 38025839 10.2147/CLEP.S427933 PMC10644870

[9] J. A. Myers , J. A. Rassen , J. J. Gagne , et al., “Effects of Adjusting for Instrumental Variables on Bias and Precision of Effect Estimates,” American Journal of Epidemiology 174 (2011): 1213–1222. 22025356 10.1093/aje/kwr364 PMC3254160

[10] B. L. DiPrete , C. J. Girman , P. Mavros , A. Breskin , and M. A. Brookhart , “Characterizing Imbalance in the Tails of the Propensity Score Distribution,” American Journal of Epidemiology 193 (2024): 389–403. 37830395 10.1093/aje/kwad200

[11] P. Ding , T. J. Vanderweele , and J. M. Robins , “Instrumental Variables as Bias Amplifiers With General Outcome and Confounding,” Biometrika 104 (2017): 291–302. 29033459 10.1093/biomet/asx009 PMC5636691

[12] J. M. Wooldridge , “Should Instrumental Variables Be Used as Matching Variables?,” Research in Economics 70 (2016): 232–237.

[13] S. M. Shortreed and A. Ertefaie , “Outcome‐Adaptive Lasso: Variable Selection for Causal Inference,” Biometrics 73 (2017): 1111–1122. 28273693 10.1111/biom.12679 PMC5591052

[14] D. Talbot , G. Lefebvre , and J. Atherton , “The Bayesian Causal Effect Estimation Algorithm,” Journal of Causal Inference 3 (2015): 207–236.

[15] X. de Luna , I. Waernbaum , and T. S. Richardson , “Covariate Selection for the Nonparametric Estimation of an Average Treatment Effect,” Biometrika 98 (2011): 861–875.

[16] J. R. Zubizarreta , “Stable Weights That Balance Covariates for Estimation With Incomplete Outcome Data,” Journal of the American Statistical Association 110 (2015): 910–922.

[17] Y. Wang and J. R. Zubizarreta , “Minimal Dispersion Approximately Balancing Weights: Asymptotic Properties and Practical Considerations,” Biometrika 107 (2019): asz050.

[18] H. Zou , “The Adaptive Lasso and Its Oracle Properties,” Journal of the American Statistical Association 101 (2006): 1418–1429.

[19] W. W. Loh and S. Vansteelandt , “Confounder Selection Strategies Targeting Stable Treatment Effect Estimators,” Statistics in Medicine 40 (2021): 607–630. 33150645 10.1002/sim.8792

[20] C. Cinelli , A. Forney , and J. Pearl , “A Crash Course in Good and Bad Controls,” Sociological Methods & Research 53 (2024): 1071–1104.

[21] E. F. Schisterman , S. R. Cole , and R. W. Platt , “Overadjustment Bias and Unnecessary Adjustment in Epidemiologic Studies,” Epidemiology 20 (2009): 488–495. 19525685 10.1097/EDE.0b013e3181a819a1 PMC2744485

[22] M. L. Petersen and M. J. van der Laan , “Causal Models and Learning From Data: Integrating Causal Modeling and Statistical Estimation,” Epidemiology 25 (2014): 418–426. 24713881 10.1097/EDE.0000000000000078 PMC4077670

[23] W. W. Loh and D. Ren , “The Unfulfilled Promise of Longitudinal Designs for Causal Inference,” Collabra: Psychology 9 (2023): 89142.

[24] S. R. Cole , R. W. Platt , E. F. Schisterman , et al., “Illustrating Bias due to Conditioning on a Collider,” International Journal of Epidemiology 39 (2010): 417–420. 19926667 10.1093/ije/dyp334 PMC2846442

[25] S. Greenland , “Quantifying Biases in Causal Models: Classical Confounding vs Collider‐Stratification Bias,” Epidemiology 14 (2003): 300–306. 12859030

[26] M. J. van der Laan and S. Rose , Targeted Learning: Causal Inference for Observational and Experimental Data (Springer, 2011).

[27] H. Bang and J. M. Robins , “Doubly Robust Estimation in Missing Data and Causal Inference Models,” Biometrics 61 (2005): 962–973. 16401269 10.1111/j.1541-0420.2005.00377.x

[28] J. M. Robins , A. Rotnitzky , and L. P. Zhao , “Estimation of Regression Coefficients When Some Regressors Are Not Always Observed,” Journal of the American Statistical Association 89 (1994): 846–866.

[29] A. Chattopadhyay , C. H. Hase , and J. R. Zubizarreta , “Balancing vs Modeling Approaches to Weighting in Practice,” Statistics in Medicine 39 (2020): 3227–3254. 32882755 10.1002/sim.8659

[30] J. R. Zubizarreta , Y. Li , K. Kim , A. Allouah , and N. Greifer , “sbw: Stable Balancing Weights for Causal Inference and Missing Data,” (2021) accessed December 29, 2023, https://cran.r‐project.org/web/packages/sbw/index.html.

[31] J. Friedman , T. Hastie , and R. Tibshirani , “Regularization Paths for Generalized Linear Models via Coordinate Descent,” Journal of Statistical Software 33 (2010): 1–22, http://www.jstatsoft.org/v33/i01/. 20808728 PMC2929880

[32] D. J. Kereiakes , R. L. Obenchain , B. L. Barber , et al., “Abciximab Provides Cost‐Effective Survival Advantage in High‐Volume Interventional Practice,” American Heart Journal 140 (2000): 603–610. 11011333 10.1067/mhj.2000.109647

[33] D. F. McCaffrey , B. A. Griffin , D. Almirall , M. E. Slaughter , R. Ramchand , and L. F. Burgette , “A Tutorial on Propensity Score Estimation for Multiple Treatments Using Generalized Boosted Models,” Statistics in Medicine 32 (2013): 3388–3414. 23508673 10.1002/sim.5753 PMC3710547

[34] F. Li and F. Li , “Propensity Score Weighting for Causal Inference With Multiple Treatments,” Annals of Applied Statistics 13 (2019): 2389–2415, https://projecteuclid.org/journals/annals‐of‐applied‐statistics/volume‐13/issue‐4/Propensity‐score‐weighting‐for‐causal‐inference‐with‐multiple‐treatments/10.1214/19‐AOAS1282.full.

[35] R. K. Crump , V. J. Hotz , G. W. Imbens , and O. A. Mitnik , “Dealing With Limited Overlap in Estimation of Average Treatment Effects,” Biometrika 96 (2009): 187–199.

[36] F. Li , K. L. Morgan , and A. M. Zaslavsky , “Balancing Covariates via Propensity Score Weighting,” Journal of the American Statistical Association 113 (2018): 390–400.

[37] H. Mao , L. Li , and T. Greene , “Propensity Score Weighting Analysis and Treatment Effect Discovery,” Statistical Methods in Medical Research 28 (2019): 2439–2454. 29921162 10.1177/0962280218781171

[38] F. Li , L. E. Thomas , and F. Li , “Addressing Extreme Propensity Scores via the Overlap Weights,” American Journal of Epidemiology 188 (2018): 250–257, https://academic.oup.com/aje/advance‐article/doi/10.1093/aje/kwy201/5090958. 10.1093/aje/kwy201 30189042

[39] L. Li and T. Greene , “A Weighting Analogue to Pair Matching in Propensity Score Analysis,” International Journal of Biostatistics 9 (2013): 215–234. 23902694 10.1515/ijb-2012-0030

[40] E. H. Kennedy , “Nonparametric Causal Effects Based on Incremental Propensity Score Interventions,” Journal of the American Statistical Association 114 (2019): 645–656.

[41] W. W. Loh and D. Ren , “The Incremental Propensity Score Approach for Diversity Science,” Advances in Methods and Practices in Psychological Science 7 (2024): 25152459241240681.

[42] A. I. Naimi , J. E. Rudolph , E. H. Kennedy , et al., “Incremental Propensity Score Effects for Time‐Fixed Exposures,” Epidemiology 32 (2021): 202–208. 33470712 10.1097/EDE.0000000000001315 PMC9040452

[43] D. Malinsky and D. Danks , “Causal Discovery Algorithms: A Practical Guide,” Philosophy Compass 13 (2018): e12470.

[44] M. L. Petersen , “Commentary: Applying a Causal Road Map in Settings With Time‐Dependent Confounding,” Epidemiology 25 (2014): 898–901. 25265135 10.1097/EDE.0000000000000178 PMC4460577

[45] O. Sofrygin , M. J. van der Laan , and R. Neugebauer , “ **Simcausal** *R* Package: Conducting Transparent and Reproducible Simulation Studies of Causal Effect Estimation With Complex Longitudinal Data,” Journal of Statistical Software 81 (2017): 1–47, http://www.jstatsoft.org/v81/i02/. 10.18637/jss.v081.i02 PMC5667661 29104515

[46] B. Y. Choi , C. Wang , and J. Gelfond , “Machine Learning Outcome Regression Improves Doubly Robust Estimation of Average Causal Effects,” Pharmacoepidemiology and Drug Safety 29 (2020): 1120–1133. 32716126 10.1002/pds.5074 PMC8098857

[47] I. J. Dahabreh , S. E. Robertson , E. J. Tchetgen , E. A. Stuart , and M. A. Hernán , “Generalizing Causal Inferences From Individuals in Randomized Trials to All Trial‐Eligible Individuals,” Biometrics 75 (2019): 685–694. 30488513 10.1111/biom.13009 PMC10938232

[48] I. J. Dahabreh , S. E. Robertson , J. A. Steingrimsson , E. A. Stuart , and M. A. Hernán , “Extending Inferences From a Randomized Trial to a New Target Population,” Statistics in Medicine 39 (2020): 1999–2014. 32253789 10.1002/sim.8426

[49] A. Chattopadhyay , E. R. Cohn , and J. R. Zubizarreta , “One‐Step Weighting to Generalize and Transport Treatment Effect Estimates to a Target Population,” American Statistician 78 (2023): 1–10.

