# Supplementary material for: Modeling Versus Balancing Approaches to Addressing Instrumental Variables in Weighting: A Comparison of the Outcome‐Adaptive Lasso, Stable Balancing Weighting, and Stable Confounder Selection
Source: Pharmacoepidemiol Drug Saf. 2025 Jun 27;34(7):e70173. doi: 10.1002/pds.70173 (PMC12203767; doi:10.1002/pds.70173)
Supplement: Supplementary file 3 — Data S3. Supporting Information. [file PDS-34-e70173-s001.docx]

**Modeling versus balancing approaches to addressing instrumental variables in weighting: a comparison of the outcome-adaptive lasso, stable balancing weighting, and stable confounder selection**

*Choi and Brookhart*

**Supplementary Material**

Supplementary Table 1. Simulation results with a sample size of 2000 when there are no correlations among the covariates $(\rho=0)$ and treatment effects are homogeneous.

| Number of | IV | Estimation | Performance measures | | | | | |
| --- | --- | --- | --- | --- | --- | --- | --- | --- |
| Covariates | Strength | Methods | Bias | MSE | R-MSE | EmpSE | AgeSE | CovRate |
| 20 | 0.5 | Target | 0.001 | 0.009 | 1.000 | 0.095 | 0.094 | 0.944 |
|  |  | Confounders | 0.000 | 0.010 | 1.171 | 0.102 | 0.103 | 0.948 |
|  |  | All covariates | 0.000 | 0.011 | 1.193 | 0.103 | 0.104 | 0.954 |
|  |  | OAL | 0.000 | 0.010 | 1.165 | 0.102 | 0.101 | 0.950 |
|  |  | SCS | 0.000 | 0.011 | 1.179 | 0.103 | 0.103 | 0.952 |
|  |  | SBW | 0.001 | 0.010 | 1.084 | 0.099 | 0.098 | 0.950 |
|  | 1.0 | Target | 0.010 | 0.009 | 1.000 | 0.096 | 0.093 | 0.938 |
|  |  | Confounders | 0.005 | 0.020 | 2.124 | 0.141 | 0.129 | 0.930 |
|  |  | All covariates | 0.004 | 0.021 | 2.205 | 0.143 | 0.131 | 0.936 |
|  |  | OAL | 0.006 | 0.017 | 1.784 | 0.129 | 0.115 | 0.918 |
|  |  | SCS | 0.006 | 0.019 | 2.068 | 0.139 | 0.123 | 0.918 |
|  |  | SBW | 0.009 | 0.013 | 1.431 | 0.115 | 0.107 | 0.926 |
| 40 | 0.5 | Target | 0.007 | 0.008 | 1.000 | 0.091 | 0.094 | 0.950 |
|  |  | Confounders | 0.007 | 0.010 | 1.183 | 0.099 | 0.103 | 0.954 |
|  |  | All covariates | 0.008 | 0.010 | 1.227 | 0.101 | 0.104 | 0.954 |
|  |  | OAL | 0.008 | 0.010 | 1.177 | 0.099 | 0.102 | 0.956 |
|  |  | SCS | 0.008 | 0.010 | 1.208 | 0.100 | 0.102 | 0.956 |
|  |  | SBW | 0.006 | 0.009 | 1.086 | 0.095 | 0.098 | 0.956 |
|  | 1.0 | Target | 0.001 | 0.008 | 1.000 | 0.090 | 0.093 | 0.960 |
|  |  | Confounders | 0.004 | 0.018 | 2.281 | 0.136 | 0.130 | 0.948 |
|  |  | All covariates | 0.006 | 0.019 | 2.368 | 0.139 | 0.135 | 0.940 |
|  |  | OAL | 0.003 | 0.017 | 2.088 | 0.130 | 0.123 | 0.942 |
|  |  | SCS | 0.004 | 0.017 | 2.152 | 0.132 | 0.124 | 0.932 |
|  |  | SBW | 0.001 | 0.012 | 1.524 | 0.111 | 0.107 | 0.950 |

Supplementary Table 2. Simulation results with a sample size of 2000 when there are strong correlations among the covariates $(\rho=0.5)$ and treatment effects are homogeneous.

| Number of | IV | Estimation | Performance measures | | | | | |
| --- | --- | --- | --- | --- | --- | --- | --- | --- |
| Covariates | Strength | Methods | Bias | MSE | R-MSE | EmpSE | AgeSE | CovRate |
| 20 | 0.5 | Target | 0.001 | 0.013 | 1.000 | 0.113 | 0.118 | 0.962 |
|  |  | Confounders | 0.000 | 0.018 | 1.412 | 0.134 | 0.132 | 0.942 |
|  |  | All covariates | 0.000 | 0.018 | 1.437 | 0.135 | 0.134 | 0.948 |
|  |  | OAL | 0.001 | 0.017 | 1.333 | 0.130 | 0.129 | 0.948 |
|  |  | SCS | 0.001 | 0.015 | 1.212 | 0.124 | 0.125 | 0.938 |
|  |  | SBW | 0.002 | 0.011 | 0.853 | 0.104 | 0.107 | 0.966 |
|  | 1.0 | Target | 0.007 | 0.018 | 1.000 | 0.135 | 0.133 | 0.948 |
|  |  | Confounders | 0.008 | 0.058 | 3.190 | 0.241 | 0.204 | 0.958 |
|  |  | All covariates | 0.008 | 0.053 | 2.896 | 0.229 | 0.204 | 0.964 |
|  |  | OAL | 0.000 | 0.035 | 1.907 | 0.186 | 0.178 | 0.954 |
|  |  | SCS | 0.006 | 0.034 | 1.868 | 0.184 | 0.163 | 0.926 |
|  |  | SBW | 0.004 | 0.014 | 0.793 | 0.120 | 0.121 | 0.944 |
| 40 | 0.5 | Target | 0.008 | 0.014 | 1.000 | 0.117 | 0.118 | 0.960 |
|  |  | Confounders | 0.005 | 0.017 | 1.217 | 0.129 | 0.132 | 0.952 |
|  |  | All covariates | 0.006 | 0.018 | 1.308 | 0.134 | 0.136 | 0.952 |
|  |  | OAL | 0.007 | 0.016 | 1.168 | 0.127 | 0.131 | 0.950 |
|  |  | SCS | 0.007 | 0.015 | 1.071 | 0.121 | 0.123 | 0.946 |
|  |  | SBW | 0.006 | 0.010 | 0.741 | 0.101 | 0.107 | 0.954 |
|  | 1.0 | Target | 0.006 | 0.020 | 1.000 | 0.143 | 0.132 | 0.946 |
|  |  | Confounders | 0.004 | 0.055 | 2.701 | 0.235 | 0.201 | 0.948 |
|  |  | All covariates | 0.006 | 0.060 | 2.940 | 0.245 | 0.211 | 0.950 |
|  |  | OAL | 0.007 | 0.048 | 2.383 | 0.220 | 0.194 | 0.952 |
|  |  | SCS | 0.005 | 0.036 | 1.768 | 0.190 | 0.155 | 0.904 |
|  |  | SBW | 0.009 | 0.017 | 0.844 | 0.131 | 0.121 | 0.928 |

Supplementary Table 3. Simulation results with a sample size of 2000 when there are no correlations among the covariates $(\rho=0)$ and treatment effects are heterogeneous.

| Number of | IV | Estimation | Performance measures | | | | | |
| --- | --- | --- | --- | --- | --- | --- | --- | --- |
| Covariates | Strength | Methods | Bias | MSE | R-MSE | EmpSE | AgeSE | CovRate |
| 20 | 0.5 | Target | 0.003 | 0.011 | 1.000 | 0.104 | 0.103 | 0.946 |
|  |  | Confounders | 0.002 | 0.013 | 1.207 | 0.115 | 0.113 | 0.954 |
|  |  | All covariates | 0.003 | 0.014 | 1.245 | 0.116 | 0.114 | 0.956 |
|  |  | OAL | 0.003 | 0.013 | 1.186 | 0.114 | 0.110 | 0.956 |
|  |  | SCS | 0.002 | 0.013 | 1.207 | 0.115 | 0.110 | 0.954 |
|  |  | SBW | 0.001 | 0.011 | 1.043 | 0.107 | 0.105 | 0.942 |
|  | 1.0 | Target | 0.010 | 0.010 | 1.000 | 0.102 | 0.100 | 0.946 |
|  |  | Confounders | 0.001 | 0.022 | 2.065 | 0.147 | 0.140 | 0.946 |
|  |  | All covariates | 0.000 | 0.022 | 2.144 | 0.150 | 0.143 | 0.944 |
|  |  | OAL | 0.003 | 0.018 | 1.737 | 0.135 | 0.125 | 0.926 |
|  |  | SCS | 0.004 | 0.020 | 1.914 | 0.142 | 0.131 | 0.926 |
|  |  | SBW | 0.008 | 0.014 | 1.334 | 0.118 | 0.114 | 0.946 |
| 40 | 0.5 | Target | 0.008 | 0.010 | 1.000 | 0.099 | 0.103 | 0.960 |
|  |  | Confounders | 0.008 | 0.012 | 1.208 | 0.109 | 0.113 | 0.958 |
|  |  | All covariates | 0.009 | 0.012 | 1.251 | 0.111 | 0.114 | 0.952 |
|  |  | OAL | 0.008 | 0.012 | 1.197 | 0.108 | 0.111 | 0.956 |
|  |  | SCS | 0.009 | 0.012 | 1.195 | 0.108 | 0.109 | 0.950 |
|  |  | SBW | 0.006 | 0.010 | 1.050 | 0.102 | 0.104 | 0.958 |
|  | 1.0 | Target | 0.001 | 0.009 | 1.000 | 0.093 | 0.100 | 0.970 |
|  |  | Confounders | 0.006 | 0.021 | 2.419 | 0.145 | 0.144 | 0.958 |
|  |  | All covariates | 0.008 | 0.022 | 2.507 | 0.147 | 0.149 | 0.954 |
|  |  | OAL | 0.006 | 0.020 | 2.259 | 0.140 | 0.135 | 0.942 |
|  |  | SCS | 0.005 | 0.018 | 2.137 | 0.136 | 0.133 | 0.938 |
|  |  | SBW | 0.001 | 0.013 | 1.495 | 0.114 | 0.114 | 0.954 |

Supplementary Table 4. Simulation results with a sample size of 2000 when there are strong correlations among the covariates $(\rho=0.5)$ and treatment effects are heterogeneous.

| Number of | IV | Estimation | Performance measures | | | | | |
| --- | --- | --- | --- | --- | --- | --- | --- | --- |
| Covariates | Strength | Methods | Bias | MSE | R-MSE | EmpSE | AgeSE | CovRate |
| 20 | 0.5 | Target | 0.008 | 0.018 | 1.000 | 0.135 | 0.147 | 0.972 |
|  |  | Confounders | 0.008 | 0.026 | 1.443 | 0.163 | 0.166 | 0.964 |
|  |  | All covariates | 0.008 | 0.027 | 1.498 | 0.166 | 0.168 | 0.960 |
|  |  | OAL | 0.009 | 0.025 | 1.338 | 0.156 | 0.160 | 0.958 |
|  |  | SCS | 0.007 | 0.020 | 1.118 | 0.143 | 0.143 | 0.940 |
|  |  | SBW | 0.004 | 0.012 | 0.646 | 0.109 | 0.118 | 0.962 |
|  | 1.0 | Target | 0.008 | 0.029 | 1.000 | 0.170 | 0.171 | 0.964 |
|  |  | Confounders | 0.002 | 0.171 | 5.930 | 0.414 | 0.272 | 0.958 |
|  |  | All covariates | 0.001 | 0.156 | 5.393 | 0.395 | 0.278 | 0.958 |
|  |  | OAL | 0.000 | 0.069 | 2.379 | 0.262 | 0.236 | 0.954 |
|  |  | SCS | 0.007 | 0.049 | 1.702 | 0.222 | 0.188 | 0.910 |
|  |  | SBW | 0.005 | 0.016 | 0.565 | 0.128 | 0.132 | 0.956 |
| 40 | 0.5 | Target | 0.010 | 0.022 | 1.000 | 0.147 | 0.150 | 0.966 |
|  |  | Confounders | 0.011 | 0.025 | 1.168 | 0.159 | 0.167 | 0.984 |
|  |  | All covariates | 0.009 | 0.028 | 1.294 | 0.168 | 0.172 | 0.976 |
|  |  | OAL | 0.011 | 0.025 | 1.162 | 0.159 | 0.167 | 0.970 |
|  |  | SCS | 0.009 | 0.019 | 0.895 | 0.139 | 0.142 | 0.968 |
|  |  | SBW | 0.007 | 0.012 | 0.540 | 0.108 | 0.118 | 0.968 |
|  | 1.0 | Target | 0.004 | 0.030 | 1.000 | 0.172 | 0.171 | 0.954 |
|  |  | Confounders | 0.003 | 0.103 | 3.483 | 0.322 | 0.265 | 0.956 |
|  |  | All covariates | 0.004 | 0.123 | 4.138 | 0.351 | 0.282 | 0.960 |
|  |  | OAL | 0.001 | 0.088 | 2.952 | 0.296 | 0.258 | 0.954 |
|  |  | SCS | 0.004 | 0.048 | 1.621 | 0.220 | 0.180 | 0.890 |
|  |  | SBW | 0.010 | 0.019 | 0.648 | 0.139 | 0.133 | 0.938 |
